# Pancreatic tissue engineering: towards a vascularized bioengineered cure for diabetes

**DOI:** 10.3389/frtra.2026.1768497

**Published:** 2026-06-08

**Authors:** Ji Ho Park, Khadijat A. Olawuyi, Neekita R. Jikaria, Jazzmyn S. Dawes, Ishak E. Dabsha, André A. Dick, Dino J. Ravnic

**Affiliations:** 1 Irvin S. Zubar Plastic Surgery Research Laboratory, Department of Surgery, Penn State College of Medicine, Hershey, PA, United States; 2 Department of Surgery, Penn State Health Milton S. Hershey Medical Center, Hershey, PA, United States; 3 Department of Biology, School of Science, Engineering and Tech, Penn State Harrisburg, Middletown, PA, United States; 4 Department of Surgery, Division of Pediatric Transplant Surgery, Seattle Children’s Hospital, Seattle, WA, United States; 5 Huck Institutes of the Life Sciences, The Pennsylvania State University, University Park, PA, United States

**Keywords:** diabetes, islet cell, pancreas, tissue engineering, transplant, vascularization

## Abstract

Type 1 diabetes results from autoimmune destruction of pancreatic β-cells, and while whole-organ pancreas and islet transplantation can restore endogenous insulin secretion, both are limited by donor scarcity, early inflammatory injury, hypoxia, and the need for lifelong immunosuppression. Pancreatic tissue engineering has therefore emerged as a strategy to generate scalable, transplantable constructs capable of reestablishing physiological glucose regulation. However, as native islets depend on a robust microvascular network for oxygen delivery, nutrients and coordinated hormone secretion, insufficient vascularization remains the dominant cause of engineered graft failure. This review provides an overview of (a) the current state of pancreas transplant, (b) strategies for vascularized and immunoprotective pancreatic constructs, (c) cell differentiation strategies beyond of pancreatic β-cells, (d) translational progress in animal and human clinical trials, (e) key challenges in clinical translation, (f) ethical and regulatory considerations, and (g) future directions for tissue engineered pancreatic constructs. Our goal is to provide the reader with a translational overview that spans biology, tissue engineering, and clinical translation.

**Impact statement:**

This translational review provides a concise review of tissue engineering efforts to date within the context of engineered pancreatic constructs. Our paper systematically and logically discusses current developments, challenges, and novel approaches in pancreatic tissue engineering. It provides a digestible starting point for understanding the various approaches and the implications of successful tissue engineering strategies that can be used to develop transplantable pancreatic constructs.

## 1 Introduction

Diabetes mellitus affects over 500 million individuals worldwide and represents a major driver of cardiovascular disease, renal failure, blindness, and premature mortality (1). Type 1 diabetes (T1D) arises from autoimmune destruction of pancreatic beta (β)-cells, while insulin-dependent type 2 diabetes (T2D) reflects progressive β-cell failure superimposed on insulin resistance (1, 2). Despite major advances in insulin delivery systems and glucose monitoring, exogenous insulin therapy fails to replicate physiologic glucose regulation, leaving patients vulnerable to hypoglycemia, hyperglycemia, and long-term complications (3).

Whole-organ pancreas and islet transplantation demonstrate that restoration of endogenous insulin secretion can normalize glycemic control and eliminate hypoglycemia unawareness (4, 5). However, both approaches are constrained by donor shortages, immune rejection, operative morbidity, and early graft loss (6, 7). As a result, pancreatic tissue engineering has emerged as a compelling alternative strategy aimed at generating transplantable, immune-protected, and scalable pancreatic constructs.

Early efforts focused primarily on the replacement of insulin-producing β-cells. However, the pancreas is not a monocellular organ. Glucose homeostasis is governed by a multicellular endocrine network composed of β-, alpha (α)-, delta (δ)-, and pancreatic polypeptide (PP) cells embedded within a richly vascularized microenvironment that enables rapid nutrient sensing and hormone exchange (8, 9). Native islet cells receive disproportionately high blood flow relative to their mass, and this vascular specialization is essential for maintaining adequate oxygenation, nutrient exchange, and tightly coupled hormone secretion (10).

Across engineered systems, failure of early and robust vascularization remains the dominant cause of graft hypoxia, apoptosis, and long-term functional decline. Experimental studies show that transplanted islet cells experience prolonged periods of low oxygen tension and reduced vascular density compared to native islets (11). Consequently, improving graft vascularization enhances insulin secretory function and survival. Thus, effective β-cell replacement will require not only the appropriate endocrine cell composition and maturation, but also the deliberate engineering of the vascular interface (12). This review critically examines the current state of pancreatic tissue engineering with emphasis on strategies that integrate multicellular endocrine biology with immunoprotective and pro-angiogenic design. We review decellularized pancreas scaffolds, stem cell-derived organoids, 3-dimensional (3D) bioprinting and fabrication technologies, encapsulation and immune-protection platforms, and clinical-stage bioartificial pancreas devices. We then focus on mechanisms to reduce immunogenicity, vascularization strategies, and translational progress, from small-animal models to human trials. Finally, we discuss remaining ethical and regulatory challenges that must be addressed to realize a durable, bioengineered cure for diabetes.

## 2 Current landscape of pancreas transplantation and limitations

**Figure 1:**
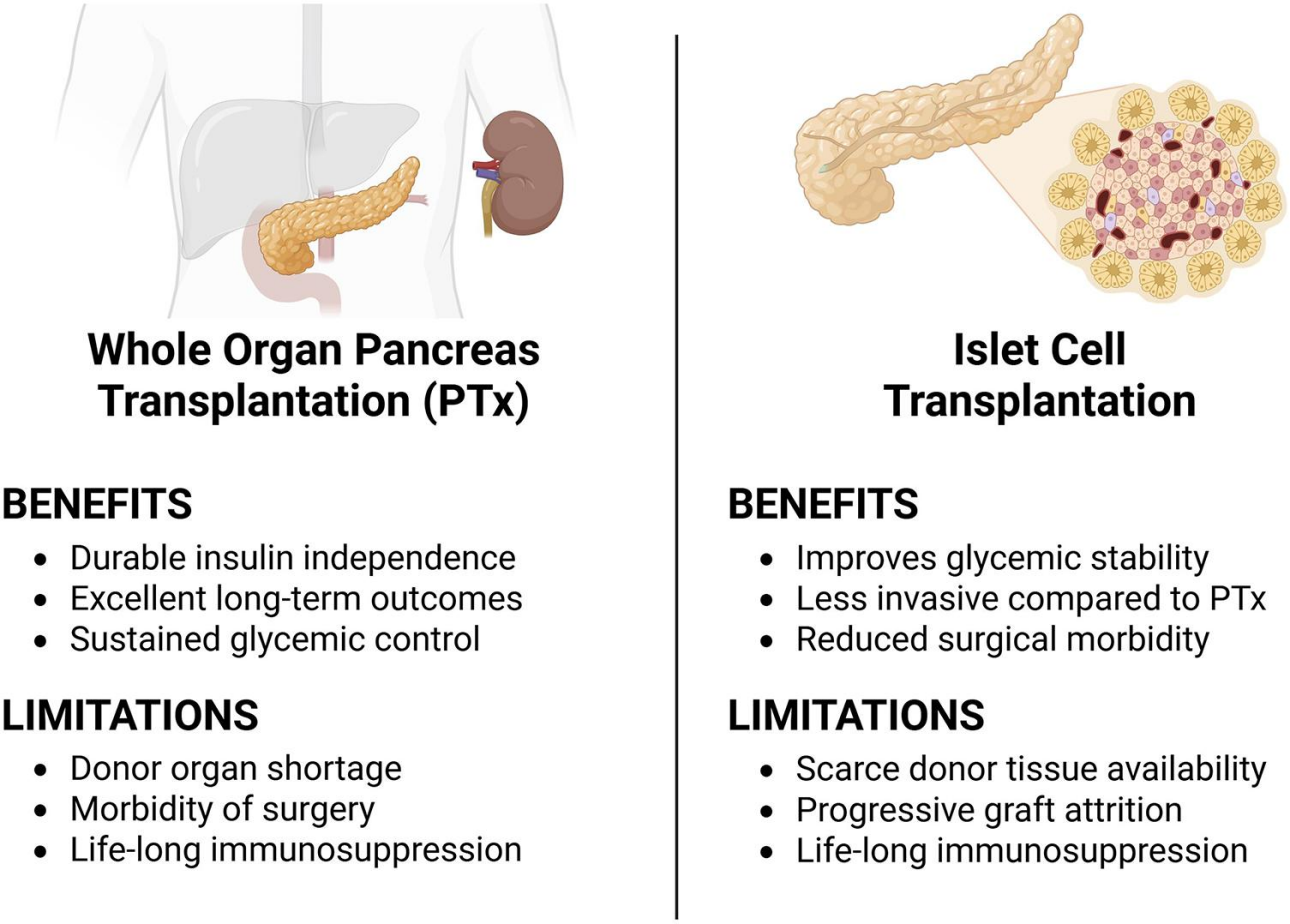
Current landscape of pancreas transplantation and their limitations. Made with BioRender.

Pancreas transplantation (whole organ and islet cell) has evolved into a recognized therapeutic option for select patients with insulin-dependent diabetes mellitus, offering the potential for durable glycemic control and improved quality of life (5–7, 13). Despite significant advances in perioperative management, surgical techniques, immunosuppressive regimen, the procedures remain constrained by challenges related to donor availability, graft survival, and long-term consequences. The following sections will critically examine the contemporary landscape of pancreas transplantation (whole organ and islet cell), highlighting prevailing practices, clinical outcomes, and the inherent limitations that continue to shape its role within modern transplant medicine (Figure 1).

### 2.1 Whole organ pancreas transplantation

Whole-organ pancreas transplantation (PTx) remains the only established therapy capable of restoring durable, insulin-independent normoglycemia in patients with complicated T1D, particularly when performed as a simultaneous pancreas–kidney (SPK) transplant in individuals with end-stage renal disease (13–15). Registry analyses from the International Pancreas Transplant Registry demonstrate that SPK offers excellent long-term outcomes, with 5- and 10-year pancreas graft function rates of approximately 73% and 56%, respectively, and parallel improvements in patient survival compared with historical cohorts managed medically (7, 13). More contemporary data show 3-year patient survival exceeding 90% and SPK pancreas graft survival >86% in the modern era, underscoring the durability of whole-organ transplantation when early graft loss is avoided (6, 14). These outcomes translate into sustained near-normal glycemic control, reductions in severe hypoglycemia, and stabilization or improvement of diabetes-related microvascular complications, particularly in appropriately selected SPK recipients (13–15).

Despite these benefits, whole-organ PTx requires major intra-abdominal surgery in a population frequently burdened by advanced diabetes complications and cardiovascular comorbidities. The operation is followed by lifelong immunosuppression, typically tacrolimus- and mycophenolate-based regimens with or without corticosteroids. This carries cumulative risks of opportunistic infection, malignancy, nephrotoxicity, and metabolic complications (16). Such adverse effects must be weighed against the anticipated gains in glycemic control and survival, especially in candidates without established renal failure (13, 15).

Perioperative morbidity after pancreas transplantation remains higher than for many other solid organs. Early “technical failures” are most often related to vascular thrombosis, hemorrhage, graft pancreatitis, anastomotic leak, and intra-abdominal sepsis, whereas later complications include vascular stenosis or pseudoaneurysm, fluid collections, ureteral or enteric leaks, chronic rejection, and recurrent infections (14, 17). Interventional radiology now plays a central role in managing many of these vascular and nonvascular complications, but graft loss and re-operation remain clinically significant risks (17).

Furthermore, whole-organ PTx is fundamentally constrained by donor availability and stringent donor selection criteria. Pancreas allograft acceptance is markedly more selective than for other solid organs, and the number of pancreata recovered that are ultimately utilized for transplantation remains insufficient to meet clinical demand, particularly for candidates awaiting SPK (18). This occurs within the broader context of a global organ shortage, where an increasing reliance on older, comorbid, or donation-after-circulatory-death donors has not fully offset the growing waitlist, and is associated with higher discard rates and fewer usable organs per donor (19). These limitations in supply, coupled with the morbidity of major surgery and lifelong immunosuppression, highlight the need for complementary strategies, such as islet cell transplantation and emerging bioengineered pancreatic tissue constructs. This would expand access to β-cell replacement beyond the small subset of patients who can undergo whole-organ pancreas transplantation.

### 2.2 Islet cell transplantation

Islet cell transplantation (ICT) has emerged as a less invasive β-cell replacement strategy for patients with brittle T1D complicated by severe hypoglycemia unawareness and glycemic lability refractory to intensive medical therapy. Unlike whole-organ pancreas transplantation, ICT involves the percutaneous intraportal infusion of isolated donor islets into the liver via the portal vein, thereby avoiding major abdominal surgery and reducing perioperative morbidity (4, 5).

The landmark Edmonton Protocol, introduced in 2000, demonstrated that steroid-free immunosuppression combined with transplantation of a sufficient islet mass could achieve short-term insulin independence in patients with T1D (5). Subsequent multicenter trials confirmed that ICT reliably restores endogenous C-peptide secretion and substantially improves glycemic stability, with marked reductions in severe hypoglycemic events even when long-term insulin independence is not sustained (4, 20, 21). Contemporary series report that approximately 40%–60% of recipients maintain insulin independence at 1 year, while over 80%–90% retain measurable graft function and hypoglycemia protection at 3–5 years (20–22).

Despite these advantages, long-term durability of insulin independence after ICT remains inferior to that of whole-organ pancreas transplantation. Progressive graft attrition occurs due to a combination of instant blood-mediated inflammatory reaction, alloimmune rejection, recurrent autoimmunity, and islet exhaustion in the relatively hypoxic hepatic microenvironment (20, 23, 24). In addition, exposure of infused islets to portal venous pressure and low oxygen tension contributes to early apoptotic loss of up to 50%–70% of transplanted islet mass within the first 48 h (25). Furthermore, like whole-organ PTx, ICT still requires lifelong systemic immunosuppression, which limits its application in otherwise healthy patients with diabetes due to risks of infection, nephrotoxicity, malignancy, and metabolic toxicity (20, 21). Moreover, donor supply remains a fundamental bottleneck, as most recipients require islets from multiple cadaveric donors to achieve insulin independence, further amplifying organ scarcity (22).

Collectively, while islet transplantation offers meaningful physiological benefits and hypoglycemia protection with reduced surgical morbidity, its limited long-term durability, dependence on scarce donor tissue, and requirement for systemic immunosuppression continue to constrain its widespread clinical utility. These limitations have driven intense interest in alternative strategies such as immunoprotective encapsulation devices, stem cell–derived β-cells, and vascularized tissue-engineered pancreatic constructs.

### 2.3 Barriers to long-term graft survival

**Figure 2:**
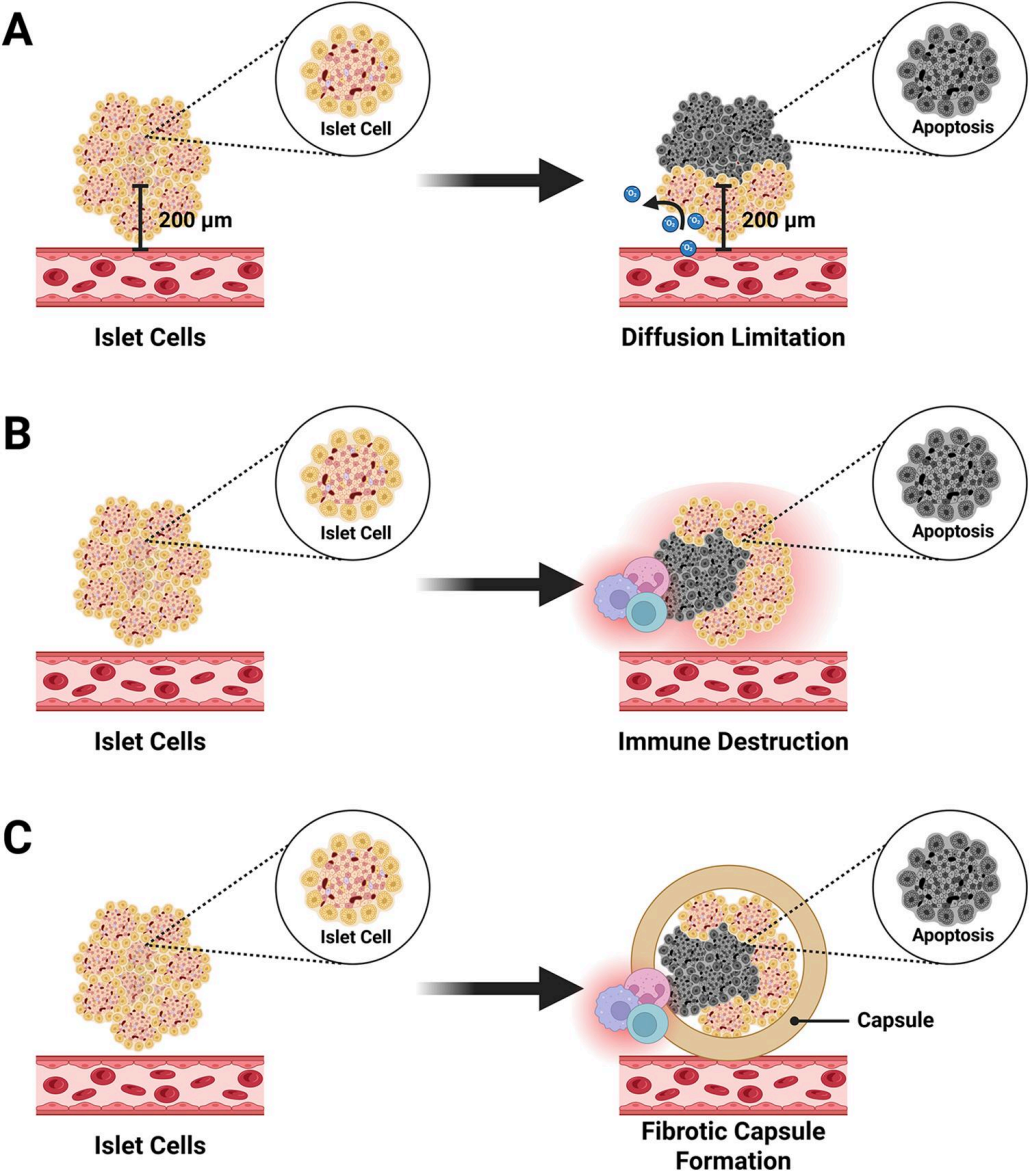
Barriers to long-term graft survival and sustained endocrine function following islet cell transplantation. Schematic illustrating three major biological constraints that limit durable graft function following islet cell transfer. **(A)** Diffusion limitations restrict oxygen and nutrient delivery to approximately 200 μm from the nearest vascular interface, resulting in central hypoxia, progressive cellular stress, and apoptotic loss of transplanted islets over time. **(B)** Immune-mediated graft destruction secondary to host immune activation drives inflammatory infiltration, direct cytotoxicity, and apoptosis. **(C)** Foreign body reaction and fibrotic capsule formation following immune-mediated graft destruction, which drives graft attrition following islet cell transplant. Made with BioRender.

Despite advances in surgical technique, immunosuppression, and perioperative management, long-term graft survival following both whole-organ PTx and ICT remains limited by a convergence of early inflammatory injury, alloimune rejection, recurrent autoimmunity, and inadequate graft vascularization (Table 1; Figure 2) (4, 25–27). These barriers result in progressive graft attrition and remain the primary obstacles to durable insulin independence.

**Table 1:** Barriers to long-term graft survival.

Barrier	Mechanism	Consequences	Reference
Instant blood-mediated inflammatory reaction	Intraportal islets trigger the activation of coagulation, complement, platelets, and innate immune cells.	>50% islet loss within 24 to 48 h before engraftment.	(25, 26)
Hypoxia or Inadequate vascularization	Disruption of native capillaries; delayed neovascularization; lower oxygen tension in transplantation sites compared to native pancreas.	β-cell apoptosis, oxidative stress, impaired insulin secretion, and graft failure.	(4, 27)
Alloimmune rejection & recurrent autoimmunity	Donor-specific antibodies, memory T/B cells, autoreactive clones, and macrophage mediated inflammation.	Progressive graft injury; recurrent diabetes after transplant despite immuno-suppression.	(4, 28–31, 33, 34, 36)
Life-long immunosuppression	Calcineurin inhibitors, anti-metabolites, and corticosteroids cause β-cell toxicity and unwanted systemic side effects.	Nephrotoxicity, hypertension, dyslipidemia, and increased risk of opportunistic infections.	(4, 16, 36)

**Figure 3:**
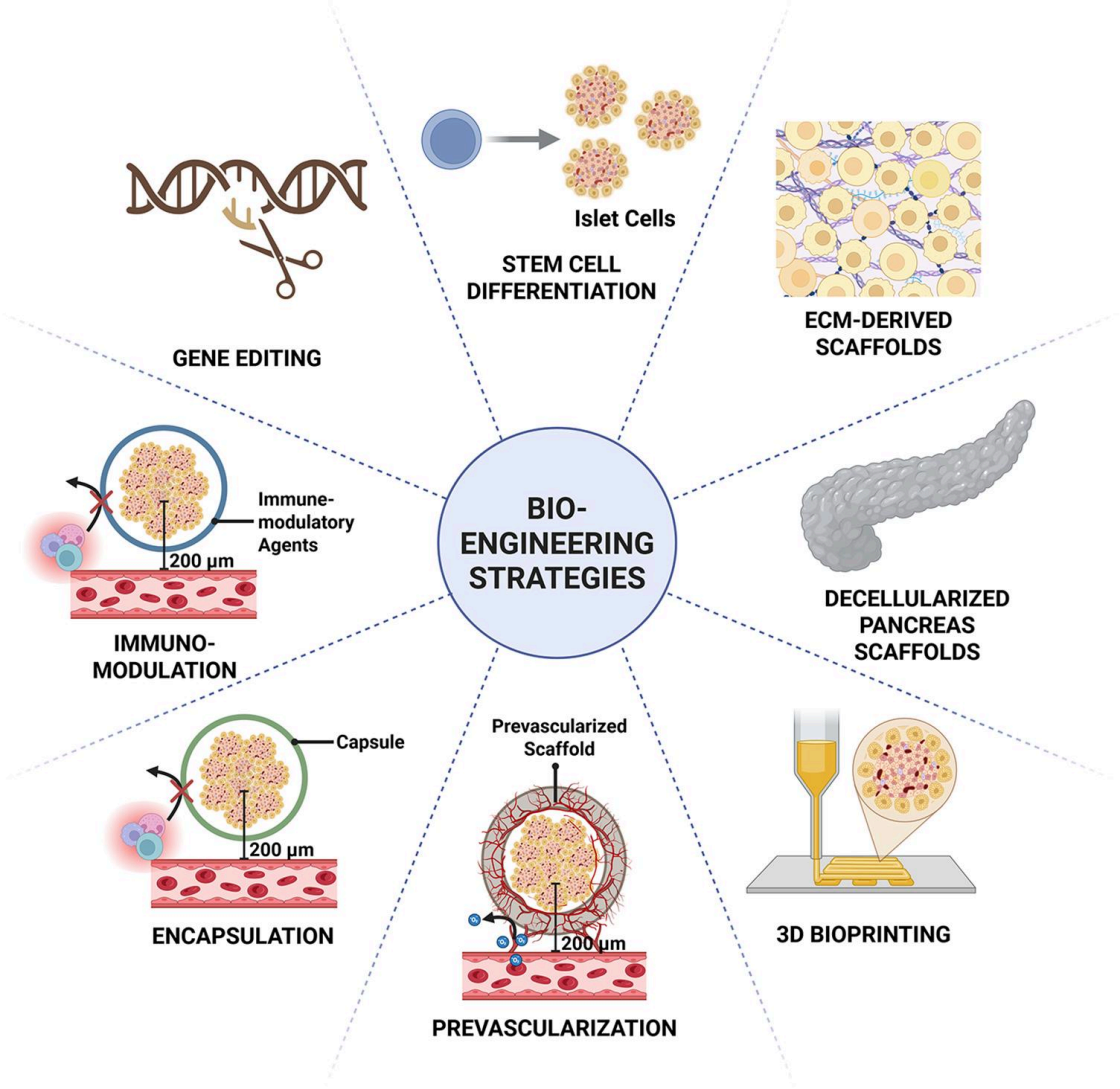
Emerging strategies to enhance islet graft survival and sustained endocrine function. Overview of complementary bioengineering and regenerative approaches aimed at overcoming key limitations in pancreatic replacement therapies. Top row illustrates scaffold-based platforms including ECM)-derived scaffolds, decellularized pancreas scaffolds, and 3D bioprinting, alongside gene-editing strategies. Bottom row highlights translational interventions including encapsulation, pre-vascularization, immunomodulation, and stem-cell differentiation. Made with BioRender.

In islet transplantation, the immediate exposure of transplanted islets to the intraportal circulation triggers the instant blood-mediated inflammatory reaction, which is characterized by activation of coagulation, complement, platelets, and innate immune cells (25, 26). This response leads to rapid ischemic and inflammatory destruction of a substantial fraction of the transplanted islet mass, often exceeding 50% within the first 24–48 h, before engraftment can occur (26). Even with modern immunosuppressive regimens, transplanted pancreatic tissue remains vulnerable to two distinct immunological treats: (1) alloimmune rejection and (2) recurrent autoimmunity (28–31). Rejection is the dominant barrier in allogeneic transplantation and occurs independently of the patient's underlying disease. This process is driven by the recognition of donor HLA molecules by host CD4^+^ and CD8^+^ *T*-cells, the activation of indirect antigen-presentation pathways, and the development of donor-specific antibodies (32). This mechanism results in progressive graft attrition despite systemic immunosuppression. In recurrent autoimmunity, the pre-existing memory *T*-cells, B-cells, and autoreactive memory clones, create an inflammatory islet microenvironment that continue to target β-cell antigens in T1D patients (33, 34). This autoimmune component has been clearly demonstrated in islet graft biopsies, demonstrating that immune tolerance to transplanted β-cells remains incomplete despite systemic immunosuppression (35). Together, alloimmune rejection and recurrent autoimmunity represent synergistic barriers to durable graft survival, underscoring the need for strategies that address both pathways in engineered pancreatic constructs

Compounding these immune-mediated injuries, the very immunosuppressive agents required to control alloimmunity and autoimmunity introduce additional metabolic and cellular stressors. Chronic exposure to calcineurin inhibitors, antimetabolites, and corticosteroids further compromises long-term graft survival through both direct β-cell toxicity and systemic adverse effects. Tacrolimus and sirolimus impair β-cell insulin secretion and promote apoptosis, while also exacerbating nephrotoxicity, hypertension, dyslipidemia, and infection risk (4, 36, 37). These toxicities limit broader application of β-cell replacement therapies in patients without renal failure and constrain repeat transplantation as graft function declines. This interdependence highlights a central paradox of current *β*-cell replacement strategies in which the immune responses that threaten graft survival then necessitate therapeutics that may compromise graft longevity

Furthermore, successful long-term graft survival is critically dependent on rapid and stable vascularization of the islets. Transplanted islets experience profound hypoxic stress immediately after infusion due to disruption of their native capillary network and delayed neovascularization within the hepatic parenchyma (4). Oxygen tension in the intraportal environment is significantly lower than in the native pancreas, impairing β-cell survival and insulin secretory capacity (4, 27). Diffusion limitations and inadequate microvascular integration promotes apoptosis, oxidative stress, and functional exhaustion, ultimately contributing to gradual graft failure (38). Altogether, these barriers highlight the critical need for engineered pancreatic constructs that provide durable immune protection and rapid, stable vascularization to achieve lasting graft function.

## 3 Engineering strategies towards transplantable pancreatic constructs

β-cells are highly receptive to microenvironment cues and destruction, such as hypoxia and vascular dysfunction, where then nutrients and oxygen restriction lead to ischemic loss and islet transplant failure. β-cell survival and proper insulin delivery is dependent on paracrine signals that maintain microenvironment communication between the surrounding extracellular matrix (ECM), insulin-producing cells, a properly functioning vasculature, and underlying immunomodulatory mechanisms (12). Therefore, current bioengineering strategies aimed at creating transplantable pancreatic constructs integrate key elements such as advanced scaffold design, scalable sources of insulin-producing cells, vascularization, and finely tuned immunomodulation (Table 2 and Figure 3).

**Table 2:** Engineering strategies for transplantable pancreatic constructs.

Strategy	Barrier Addressed	Rationale	Outcome	References
Decellularized pancreas scaffolds	Lack of native ECM vascular microarchitecture	Biocompatible platform to replicate the natural pancreatic microenvironment; maintenance of structural integrity and vascular support.	Preservation of ECM and vascular architecture; reduced immunogenicity; recellularization.	(37–41, 43, 102)
Stem cell-derived β-cells and islet organoids	ESC ethical concerns; immune rejection; scalability; functional maturity of iPSCs-derived β-cells	Self-renewal and developmental plasticity; differentiation potential; precision medicine.	Smart biogel development; integration of vascularization; creation of 3D organoid systems.	(48–55, 57–60, 151)
3D bioprinting & fabrication technologies	Replicating native islet vascular architecture; central necrosis and poor diffusion	Enables precise spatial placement of critical peri-islet microenvironment elements; tissue-specific bioinks; integration of vascular conduits and customized architectures.	Improved β-cell functionality; enhanced vascularization and viability; controlled mechanical and cellular integration; architectural precision.	(62–66, 106)
Angiogenic growth factor delivery	Insufficient vascularization after islet transplantation; significant ischemia; hypoxic injury and poor graft survival	Controlled and/or sustained delivery of growth factors (e.g., VEGF) accelerate host angiogenic responses.	Enhanced vascularization and graft survival; Improved revascularization of biomaterials.	(67–71)
EC and MSC co-transplantation	Delayed or insufficient vascularization; fibrotic encapsulation around grafts; limited inosculation with host circulation	Accelerates vascular network formation, mimics the native pancreatic microenvironment for better engraftment. immunomodulatory and anti-fibrotic effects.	Reduces fibrosis and promotes chimeric vessel formation, better graft survival.	(73, 74)
Pre-vascularized implantation constructs	Diffusion distance; delayed vascularization; hypoxia	Development of a pre-vascularized niche prior to islet implantation, ensuring adequate blood supply and oxygenation.	Robust vascularization; improved graft survival; long-term diabetes reversal without systemic immunosuppression.	(75–77)
Vasculogenic hydrogelsDiffusion distance; delayed vascularization; hypoxia		Functionalized hydrogels with angiogenic cues (e.g., VEGF, FGF) to promote host vessel ingrowth around grafts.	Enhanced vascular density; improved oxygen/nutrient delivery; prolonged islet function in rodent and large animal models.	(78, 79)
ECM-guided angiogenesis	Lack of native ECM signals in synthetic scaffolds and subsequent delayed vascularization; hypoxia	Incorporation of ECM proteins (collagen, laminins, fibronectin) mimics native pancreatic microenvironment, guiding EC behavior.	Development of organized capillary networks; improved islet survival; enhanced physiologic insulin secretion compared to inert materials.	(61, 74)
Alginate-based microcapsules	Fibrotic overgrowth and Immune rejection	Semi-permeable alginate shell allows nutrient and hormone diffusion but blocks immune components; chemical modifications reduce fibrosis; vascularized sites offset diffusion distance.	Reduction in fibrosis and maintenance of insulin secretion for up to 6 months in early NHP studies.	(81–84)
Zwitterionic hydrogelsFibrotic overgrowth and protein absorption, immune rejection		Net-neutral, hydrated surfaces resist non-specific protein binding and cell adhesion; preservation of peri-capsular space for neovascularization.	Reduction of cellular overgrowth in mice, dogs, pigs; improved long-term graft function without immunosuppression; maintenance of immune protection while supporting vascularization of device.	(86, 87)
Conformal surface coatings	Diffusion distance and transplant volume	Ultrathin PEG hydrogel directly coats islet cells, minimizing physical barrier and preserving vascular access.	Coated islets restore euglycemia in mice with normal vascularization without systemic immunosuppression.	(88, 89)
Immunomodulation	Autoimmune and alloimmune rejection	Local delivery of immunosuppressants or immune-regulatory ligands; co-transplantation of accessory cells; porous scaffolds allow full vascularization.	Suppressed immune infiltration, improved engraftment, and glycemic control in animal studies.	(90–93)

### 3.1 Decellularized extracellular matrix-based scaffolds

Recent advances in biomaterials allowed for the development of synthetic scaffolds for tissue engineering. However, the successful pancreatic organ regeneration requires ideal scaffold material to mimic the tissue-specific ECM and microvascular architecture that help guide islet organization, survival, and insulin secretory function. The vascular basement membrane and ECM help manufacture islet architecture and functionality, therefore bioengineered scaffold designs should reflect these same properties and mechanisms to stimulate vascular ingrowth and β-cell survival (39, 40). Specifically, decellularized pancreatic scaffolds are being increasingly utilized as they preserve the native ECM and vascular architecture while providing a biocompatible platform for insulin-producing cells and endothelial cells (ECs). This provides researchers with a regenerative platform that supports the functional reconstruction and integration of pancreatic tissue via structural and biochemical cues that guide proper insulin secretion, with preserved vascular networks to stimulate angiogenesis and reduced immunogenicity. The effects of incorporating live, functional cells within a decellularized construct, also known as recellularization, were found to maintain structural integrity while stimulating insulin secretion (41). Most recellularization involves induced pluripotent stem cells (iPSCs) or mesenchymal stem cells (MSC), and pancreatic progenitor cells embedded within scaffolds to help restore endocrine functionality, while many also incorporate ECs for enhanced vascularization and insulin delivery. These natural constructs can be re-functionalized using the patient's own MSCs, that were able to bypass immune rejection, lower blood glucose levels, and restore endocrine and exocrine function.

Additionally, vascularization remains a key limiting factor in tissue regeneration. Hao et al. demonstrated the angiogenic potential of decellularized pancreatic ECM derived hydrogel with increased adhesion and proliferation of ECs and upregulation of proangiogenic genes such as MMP2, VEGF, and PAR1 when compared to conventional scaffolds (42). Others employ decellularized scaffolds to dissect their translatable utility in β-cell survival and vascularization (antidiabetic effects), highlighting that while they may retain ECM architecture and low immunogenicity, the scaffolds still encounter structural variability and limited clinical translation (43, 44).

Fibrotic encapsulation is a well-recognized limitation in many synthetic islet delivery devices and polymeric scaffolds. In fact, studies have shown that implant shape, size, or architecture can independently modulate foreign body response and fibrotic encapsulation, even when material chemistry is similar (45, 46). As a result, efforts at mitigating this foreign-body response remains a central design target in various bioengineering encapsulation strategies (47). In contrast, decellularized ECM scaffolds retain native biochemical signals and immunomodulatory components. Thus, these decellularized ECM scaffolds typically elicit lower fibrogenic, foreign body responses compared to synthetic delivery devices and polymeric scaffolds. When fibrosis is observed in dECM, it is often linked to residual detergent or suboptimal decellularization (48). Thus, biologic, decellularized ECM often integrates more favorably than purely synthetic analogues.

Beyond the pancreas, emerging studies demonstrate the feasibility of alternative ECM sources, including decellularized kidney (49), liver (50, 51), spleen (52, 53), lung (52, 53), and placenta (54, 55) for the development of organ-derived bioactive scaffolds. Similiar to decellularized pancreatic scaffolds, these alternative ECM sources have been shown to retain native biochemical cues and vascular architecture and can support various cell types. These alternative sources of ECM are currently being explored for cross-organ applications, including pancreatic tissue engineering and vascularized endocrine constructs. Specifically, recent studies on lung-derived decellularized ECM platforms have demonstrated the capacity to preserve an extensive vascular architecture. Efforts at reendothelialization have achieved approximately 75% relative vascular coverage, stable barrier function, and patency following orthotopic transplantation, demonstrating its potential as a pre-vascularized endocrine platform (53). Notably, Bogomolova and colleagues compared decellularized ECM from porcine lung and pancreatic tissue as potential islet platforms *in vitro*. They found that on day 7, islets cultured on decellularized lung matrix maintained 95% viability, which was significantly higher than controls (60%) and the pancreatic matrix (83%). This suggests that alternative organ-derived ECM sources could be repurposed for the development of pancreatic constructs (56). Together, these findings suggest that alternative organ-derived ECM platforms may be strategically repurposed for development of vascularized endocrine constructs for pancreatic bioengineering.

### 3.2 Emerging strategies for β-cell replacement

Advances in β-cell replacement are essential for the development of effective therapies for diabetes. The current progress is in two main areas: (1) stem-cell-derived β-cells that are supported by organoid platforms that mimic the structure of human islets and (2) xenogeneic β-cell sources that use engineered spheroid systems to improve the survival and function of grafts. These strategies demonstrate the influence of human-engineered constructs and enhanced porcine spheroids on the advancement of future β-cell therapies.

#### 3.2.1 Stem cell-derived β-cells and islet organoids

Human pluripotent stem cells (hPSCs), including embryonic stem cells (ESCs) and iPSCs, have been instrumental in the creation of scalable, insulin-producing cells and relevant pancreatic endocrine lineages. ESCs clinical usage is minimal, as ethical concerns overshadow their regenerative potential, so, researchers have increasingly utilized iPSCs (57–59). Irrespective of their source, hPSCs retain significant utility within diabetes and regenerative medicine research as they offer self-renewal abilities, developmental plasticity, and offer patients a personalized therapeutic modality (60, 61). Researchers may utilize these hPSCs to differentiate toward definitive endoderm layers including the pancreas, liver, and gut, through influencing key differentiation factors such as Fibroblast growth factor (FGF)-10, retinoic acid for posterior foregut, Pancreatic and duodenal homeobox 1 (Pdx1) and NK6 homeobox 1 (Nkx6.1) for pancreatic progenitors, Notch inhibition and Epidermal Growth Factor for endocrine commitment while also assessing endocrine markers of key cells such as Insulin (β-cells), Glucagon (α-cells), Somatostatin (δ-cells) (62–66). These differentiation abilities highlight their utilization in creating cell therapies for diabetic conditions, in-depth disease modeling of β-cell dynamics, and drug screening. iPSC-derived β-cells are at the forefront of regenerative approaches to bioengineer pancreatic tissues for diabetic therapies or ischemic conditions. While iPSC-derived β-cells offer significant potential, they still encounter hurdles to clinical implementation as they have limited functional maturity with many resembling fetal β-cells rather than adult, immune rejection, and scalability restraints (67–69). To address these concerns and advance the clinical potential of iPSCs-derived β-cells researchers have moved to create smart biomaterials, hydrogels dynamically interact with the microenvironment to curb excess immunoreaction while leveraging pro-regenerative and pro-angiogenic mechanisms (70, 71). These efforts have also integrated vascularization, where a pre-vascularized construct or a co-culture with ECs provide enhance engraftment and oxygen delivery to the site.

Building on these advances in improving graft integration, researchers have also turned to chemical reprogramming as an alternative approach for generating iPSC lines capable of producing more mature islet-like cells. Instead of relying on the conventional method of forcing somatic cells to overexpress reprogramming transcription factors, chemically induced PSCs (CiPSCs) use defined small-molecule compounds that are easily manufactured, non–genome-integrating, highly controllable, and compatible for large-scale production. CiPSCs’ clinically suitable properties have allowed for their application in generating islet-like cell clusters, which demonstrate enhanced functional maturity when compared with previous attempts using traditional hPSC-derived islet cells. By incorporating pancreatic lineage reporter systems, researchers have improved the differentiation process to yield CiPSC-derived islets with transcriptomic identity, endocrine composition, and insulin secretory function that closely resemble native human islets. The impact of this approach was demonstrated through the transplantation of CiPSC-derived islets into individuals with T1D under immunosuppression, resulting in sustained insulin independence and improved glycemic control (72).

In addition to these efforts to better recapitulate the multi-cellular pancreatic microenvironment, researchers have also created 3D culture systems or organoids. These organoids allow for self-organization of vital pancreatic cellular elements such as β, α, δ cells (endocrine), ductal and acinar cells (exocrine), and supporting stomal/vascular cells (pericytes/ECs) (65, 73, 74).

Chimeric spheroids have been explored as a way to better understand the multicellular pancreatic environment. Recent work on stem cell–derived islets has focused on reaggregation approaches, where immature β-like cells are broken apart and then reformed into islet-sized clusters. These enriched β-clusters (eBCs) show many features of mature β-cells, including stronger insulin secretion, better calcium signaling, and improved mitochondrial activity. This shows that allowing endocrine cells to cluster naturally can help guide them to maturation (75). Other studies have created smaller chimeric spheroids that mix islet cells with other cell types. When mesenchymal stromal cells (MSCs) are added, research showed that islet viability is strengthened due to the protective layer that the MSCs add. However, increasing the amount of MSCs in the co-cultured mix and increasing the spheroid size has led to a decline in β-cell function. These chimeric spheroids show poor glucose-stimulated insulin secretion because the addition of MSCs disrupts the islet structure enough to reduce insulin secretion (76). In contrast, co-culturing islets with endothelial cells (ECs) tend to improve function. When islet clusters are grown with human umbilical vein endothelial cells, they form small vascular networks that support the islets, increase their survival, and improve insulin secretion in response to glucose. Unlike MSCs, ECs provide ECM components and paracrine signals that activate pathways like integrin-β1, which help regulate insulin production, calcium entry, and insulin release. In these vascularized organoid models, the presence of endothelial cells boosts β-cell function.

Overall, iPSCs-derived cells and organoid systems offer revolutionary outlooks in creating an unlimited source of patient-specific treatments combating diabetic related conditions while avoiding ethical concerns presented with the use of ESCs.

#### 3.2.2 Alternative xenogeneic β-cells sources

Beyond human donor islets and pluripotent stem cell constructs, which aim to mimic the multicellular pancreatic niche, xenogeneic β-cells are emerging as a relevant alternative source. One notable strategy involves neonatal porcine islets, which are islet cell clusters taken from newborn pig pancreata that can be dispersed and reaggregated into designed shapes. When these neonatal pig islets are coupled with human cord blood derived blood outgrowth endothelial cells, they produce pre-vascularized 3D spheroids that improve early graft survival and time to functional engraftment. The spheroid architecture provides improved survivability at transplanting, upregulates protective porcine stress response genes, enhances production of proangiogenic factors, and yields larger intragraft vascular density. These findings suggest that pig islets are a practical xenogeneic source, and that combining them with supportive human cells or proangiogenic cues might reduce early ischemia and inflammatory loss (77). The field has also evolved from preclinical studies to early human translation utilizing genetically altered swine organs. Since pigs breed rapidly, have large litters, and can be size matched to humans when successfully engineered, organs such as lung, heart, and kidney have been implanted in exploratory or compassionate use contexts. By eliminating major xenoantigens, adding human protective transgenes, regulating cellular immunity, and performing organ level and *ex vivo* therapies coupled with surgical immunomodulation, source pigs are being prepared for rigorous preclinical validation and tiered clinical testing. These developments directly inform β-cell sourcing tactics. Genetically modified pigs and engineered porcine islet structures, such as pre-vascularized newborn swine islet spheroids, offer a scalable near-term approach to deliver functioning xenogeneic β-cells. Integrating genetic safeguards with cell level engineering and delivery technologies gives a feasible avenue for implementing swine β-cell therapies alongside stem cell and bioprinted alternatives (78). Recent advances in porcine genome engineering have markedly improved the immunologic compatibility and functional potential of xenogeneic β-cell sources. Modern gene-edited porcine donors frequently incorporate triple-knockout modifications targeting GGTA1, CMAH, and B4GALNT2 to eliminate key xenoantigens responsible for antibody-mediated rejection (79–82). Parallel human complement-regulatory transgenes such as CD46, CD55, and CD59 are often incorporated to further modulate immune response by mitigating the complement-mediated injury and antibody triggered cytotoxicity (83, 84). Recent advances in immune-checkpoint engineering, such as the enforced expression of PD-L1 or other inhibitory ligands, allow for attenuation of T-cell mediated xenoreactivity to improve long-term graft acceptance (85, 86). Other gene edits aim to reduce early coagulation-mediated loss of islet cells following intravascular exposure via thromboregulatory edits, in which human thrombomodulin or endothelial protein C receptor is introduced (87). These modifications reduce hyperacute rejection and prolong xenograft survival in preclinical models. Moreover, these gene-edited porcine islets can be integrated with encapsulation platforms, conformal coatings, or vascularized implantation systems to create a multi-layered immune protection strategy. The convergence of genetic engineering and biomaterial-based immunomodulation represents a synergistic path forward in xenogeneic β-cell therapy. Together, these integrated genetic innovations underscore the expanding clinical relevance of gene-edited porcine *β*-cell sources for pancreatic tissue engineering.

### 3.3 3-dimensional (3D) bioprinting and fabrication technologies

3D bioprinting specifically enables controlled placement of β-cells, ECs, pericytes, stromal cells, and ECM components within a pre-defined architecture that more closely mimics the native islet-vascular units. Using extrusion or microfluidic-associated bioprinting, bioinks can be patterned into porous networks that support perfusion while maintaining high cell viability. Tissue-specific bioinks incorporating pancreatic decellularized ECM (pd-ECM) have been particularly effective in preserving *β*-cell phenotype and glucose responsiveness. Kim et al. developed a pd-ECM based bioink to print islet-laden constructs. On *in vitro* analysis, cell culture in the pd-ECM matrix increased expression of pancreatic markers and improved glucose-stimulated insulin secretion compared with generic hydrogels. Furthermore, co-culture with ECs reduced central necrosis in these 3D constructs (88).

Recent work has extended this approach by combining pd-ECM with photocrosslinkable polymers such as hyaluronic acid methacrylate or alginate to control stiffness, print fidelity, and transport properties. Wang et al. utilized hyaluronic acid methacrylate/pd-ECM hydrogels to strategically print pancreatic islet organoids that maintained high viability while restoring normoglycemia in diabetic mice for up to 12-weeks. Additionally, they found robust CD31^+^ neovascularization throughout the graft, demonstrating the of these engineered matrices to support endocrine function while allowing for vascular ingrowth (89). Likewise, alginate-pd-ECM bioinks optimized for human islet cell printing allowed for development of constructs that sustained insulin secretion and supported effective nutrient and drug effusion *in vitro*, pointing towards scalable manufacturing of patient-specific grafts (90).

Beyond the bulk bioink composition, 3D bioprinting allows for explicit patterning of vascular conduits alongside endocrine compartments. Idaszek et al. created alginate-based, tissue-specific bioinks that enabled printing of intertwined pancreatic and vascular structures. The porcine islets embedded within a pancreas-specific bioink adjacent to human umbilical vein endothelial cells and human MSCs-laden filaments were able to form vessel-like networks while retaining glucose-responsive insulin secretion, representing a step towards architecturally defined vascularized pancreas grafts (91). Moreover, Kim and colleagues successfully bioprinted “bespoke” islet niches using pd-ECM and basement membrane proteins arranged to mimic the peri-islet microenvironment. These constructs promoted intimate islet-capillary juxtaposition and enhanced revascularization *in vivo*, demonstrating that geometric guidance can be leveraged to reconstruct physiological islet-vessel relationships (92).

Multicellular printing strategies are increasingly integrating stem cell-derived endocrine cells with vascular and stromal components. Edri et al. used advanced 3D bioprinting to generate multi-cellular, stem cell-derived vascularized pancreatic constructs in which endothelial networks interconnected with endocrine clusters, improving oxygenation, and coordinated insulin secretion (93). Collectively, these studies highlight 3D bioprinting as a powerful platform to bioengineer vascularized pancreatic grafts in which endocrine, stromal, and vascular elements are co-organized, providing a bridge between *in vitro* organoid biology and fully implantable bioengineered organs.

### 3.4 Vascularization strategies

The success of endocrine pancreatic construct is fundamentally constrained by its ability to rapidly establish a dense, functional microvascular network. Thus, modern pancreatic tissue engineering strategies place vascularization at the center of construct design.

#### 3.4.1 Angiogenic growth factor delivery

One of the most widely explored strategies involves the controlled delivery of angiogenic growth factors to accelerate host-derived neovascularization. Vascular endothelial growth factor (VEGF) is the dominant driver of EC migration, proliferation, and capillary sprouting (94, 95). Native islets upregulate VEGF expression in response to hypoxia, but this response is often insufficient to overcome the profound ischemia that follows transplantation.

Localized VEGF delivery from biomaterial scaffolds, including collagen, fibrin, alginate, silk, fibroin, and polyethylene glycol (PEG)-based matrices have been shown to increase vascularization and reduce hypoxic injury. Mao et al. fabricated macro-porous silk fibroin scaffolds that released heparin, allowing for sequestration of endogenous VEGF and enhancing VEGF receptor 2 signaling. Co-transplantation of islets with this scaffold in diabetic mice accelerated islet vascularization and improved long-term graft survival compared to non-heparinized scaffolds or islets alone (96, 97). Another strategy is to develop hydrogels incorporated with proangiogenic factors. Phelps et al. examined biofunctionalized PEG-maleimide hydrogels incorporated with VEGF. When transplanted into the small bowel mesentery of rats, they found that the islets showed robust graft attachment and revascularization as early as 4 weeks. In addition, the incorporation of VEGF led to a substantively greater vascularization response compared to hydrogels without VEGF (98).

#### 3.4.2 Endothelial cell and mesenchymal stem cell co-transplantation

An alternative strategy is the direct incorporation of vessel-forming cells into engineered pancreatic constructs. Co-transplantation of islets with ECs, endothelial progenitor cells, and MSCs have repeatedly shown to accelerate inosculation with the host circulation, increase vessel density, and improve graft survival (99, 101). Fransson et al. examined co-transplantation of human islets with luciferase-GFP-transduced MSCs into immunodeficient mice. They found that MSCs not only reduced fibrotic encapsulation around the grafts, but it also promoted the migration of ECs from islets to the host tissue, forming chimeric blood vessels (100). Recent studies have examined co-transplantation of islet cells alongside multiple cell types. Zacharovová et al. Examined co-transplantation of islet cells with adipose-derived MSCs (adMSCs) alone compared to islet cells with adMSCs and ECs. Similar to other studies, they found that co-transplantation of islets and adMSCs demonstrated preservation of islet morphology and viability with significant vascularization within the decellularized pancreatic scaffold. However, when islet cells were co-transplanted with both adMSCs and ECs, they found further improvement in vascular network density and complexity compared to adMSCs alone. The presence of ECs appeared to accelerate the formation of functional microvasculature, suggesting a synergistic angiogenic effect (101).

#### 3.4.3 Pre-vascularized implantation constructs

Instead of attempting to establish vascular constructs *de novo* following implantation, the use of pre-vascularized niches seeks to establish a mature microvascular bed prior to endocrine cell delivery. Subcutaneous tissue is normally poorly perfused and hostile to islet survival. Pre-vascularization strategies fall into three conceptual categories: (1) Deviceless angiogenic subcutaneous priming, (2) chamber-based or scaffold-based revascularization, and (3) implantable NICHE devices with local immunomodulation.

Biomaterial-induced subcutaneous priming leverages controlled foreign-body responses to generate a dense, microvascular bed prior to islet cell implantation. Pepper et al. Demonstrated that transient placement of a vascular access catheter can create a device-less, pre-vascularized subcutaneous niche that supports islet engraftment in murine models (102). This demonstrated that angiogenic conditioning of subcutaneous tracks can transform poorly perfused anatomical sites into permissive endocrine niches without permanent device implantation.

Various studies have examined the utilization of pre-vascularized tissue-engineered chambers as a strategy to improve vascularization of transplanted islets (103–105). Chamber-based pre-vascularization involves implantation of an empty chamber to induce host-driven neovascularization. Utilization of a staged pre-vascularization approach led to the development of robust microvascular networks and improved engraftment of transplanted islets (103, 105). Furthermore, these islets offered improved glycemic control and glucose tolerance compared to non-pre-vascularized controls (104–106).

Lastly, Grattoni and colleagues developed a NICHE device that integrates a pre-vascularized implantable cell reservoir with a separate immunosuppressant reservoir for localized immunosuppressive delivery (107). This platform enables site-specific immune modulation while also supporting vascular ingrowth promoting long-term islet survival and reducing reliance on systemic immunosuppression. The NICHE device will be further discussed in later sections of the paper (see Section 3.6).

These principles have now been translated clinically, as demonstrated by Sernova Pouch System (see Section 5.2.2), and the Diabetes Research Institute Omental BioHub (see Section 5.3.1) that will be discussed in later sections of this paper.

#### 3.4.4 Utilization of vasculogenic hydrogels and ECM guided angiogenesis

Engineered hydrogels that actively instruct vascular growth form a major class of vascularization strategies. Modulation of hydrogel porosity, stiffness, and bioactive ligands have shown to support EC migration, lumen formation, and vessel stabilization (108–110). In the context of transplantable pancreatic constructs, the utilization of pd-ECM hydrogels allow for enhancement of β cell function and angiogenesis following implantation (88, 101). It is thought that pd-ECM retain native basement membrane proteins, integrin-binding motifs, and growth factor-binding domains that allow for preservation of morphology and rich vascularization.

Despite major advances in promoting rapid and stable graft perfusion, vascularization alone cannot overcome the persistent challenge of immune rejection. Even well-vascularized constructs remain susceptible to alloimmune, and autoimmune destruction once exposed to the host's immune surveillance.

### 3.5 Encapsulation strategies

Encapsulation approaches aim to protect transplanted islets from both autoimmune destruction and alloimmune rejection, while preserving diffusion of oxygen, nutrients, and insulin. Encapsulation platforms can be broadly categorized into: (1) alginate-based microcapsules, (2) zwitterionic hydrogels, (3) conformal surface coatings, and (4) immunomodulatory scaffolds.

Alginate microencapsulation remains the archetypal immunoisolation strategy for islet transplantation (111–113). Conventionally, islets are suspended in alginate and gelled into spherical microcapsules cross-linked with divalent cations, sometimes with a polycation overcoat. The semi-permeable shell permits diffusion of glucose, oxygen, and insulin but excludes immune cells. Depending on pore size, the shell may also exclude complement and large antibodies. Early large-animal work showed that alginate macro- and microencapsulation could correct diabetes in primates using xenogeneic pig islets for up to six months without systemic immunosuppression, supporting the feasibility of alginate as a barrier material for immunoisolation (111).

More recent formulations emphasize chemically modified alginates and extrahepatic, richly vascularized implant sites to reduce foreign-body reactions while decreasing diffusion distances. Bochenek et al. identified alginate derivatives that minimized pericapsular fibrotic overgrowth in a combinatorial screen. The new alginate derivative was then used to encapsulate allogeneic islets and transplanted into the omental bursa of macaques (112). The omental bursa provided highly vascular, retrievable bed allowing for rapid vascularization of the capsule (114). offsetting the diffusion distance created by the capsule themselves. Following implantation, these grafts maintained glucose-responsive insulin secretion for four months without immunosuppression.

Furthermore, alginate microcapsules are also being combined with local chemokine delivery to modulate the immune infiltrate without sacrificing vascular integration (112). Alagpulinsa et al. co-encapsulated human stem cell-derived β-cells with chemokine CXCL12 in alginate microcapsules. When implanted in immunocompetent diabetic mice, these capsules prolonged graft survival and function without systemic immunosuppression (113).

These studies illustrate the central trade-off to alginate systems; thick, highly cross-linked shells and fibrotic overgrowth protect against immunity but have the propensity to increase diffusion distance, resulting in hypoxic stress. This limitation could be partially overcome by combined utilization of highly vascularized implantation beds (i.e., omentum).

Zwitterionic hydrogels present a net-neutral, highly hydrated surface that strongly resists non-specific protein absorption and cell adhesion (115). Liu et al. synthesized sulfobetaine- and carboxybetaine-modified alginates and showed that these zwitterionic alginates markedly reduce cellular overgrowth around the microcapsules in mice, dogs, and pigs. When utilized to encapsulate islets and transplanted into diabetic mice, the zwitterionic alginate microcapsules improved long-term graft function compared to unmodified alginate (116). By limiting fibrotic encapsulation, these materials preserved a thin pericapsular space where neovascularization could occur and remain functional, effectively decoupling immune protection from the progressive loss of vascular access that plagues conventional capsules.

Beyond the bulk gels, zwitterionic chemistries have been integrated into thin-film and device architecture designed to maximize host-device vascular interface while maintaining low. For example, zwitterionically modified hydrogels in microdevice formats have been used to encapsulate β-cells in thick constructs without severe fibrosis, enabling the pre-vascularization of the device surface prior to cell loading, improving oxygenation across the device (117). Although these macrodevices continue to face intrinsic diffusion limitations; their zwitterionic surfaces support a more stable microvascular network, which is critical for thick grafts, or grafts with high cell burden.

An alternative strategy to overcoming immune reactivity while also mitigating diffusion limitations is utilization of conformal coatings. Conformal coating minimizes the physical barrier between the islets and the host vasculature by deposition of an ultrathin, continuous hydrogel layers directly onto the islet surface, rather than entrapping the islet in a large capsule (118). This dramatically reduces the transplant volume and diffusion distances, preserving near-native access to host microvasculature while still providing a controllable interface for immune modulation (118, 119).

Tomei et al. developed a microfluidic process to conformally coat islets with a thin PEG-based hydrogel and systematically optimized coating thickness and mechanical properties. In diabetic mouse models, conformally coated islets maintained a normal glucose-stimulated insulin secretion *in vitro* and restored long-term euglycemia *in vivo* with revascularization that was comparable to naked islets. This demonstrates that the thin coating does not impede vascular integration with host tissue (118).

Stock et al. extended this approach of stem cell-derived islets and fully MHC-mismatched recipients. Conformal PEG coatings were applied to stem cell-derived islets and transplanted into a confined, well-vascularized site. In this setting, conformally coated stem cell-derived islets achieved long-term diabetes reversal in allogeneic mice without systemic immunosuppression. This demonstrates that a thin PEG coating is sufficient for immune protection while allowing for development of robust microvascular integration within host tissue (119).

### 3.6 Immunomodulation strategies

An alternative option to autoimmune and alloimmune responses is to develop immunomodulatory scaffolds and devices that create a pro-tolerogenic microenvironment around the graft. This allows for direct host-graft contact, which allows for full vascularization of the graft. These platforms typically incorporate local delivery of small-molecule drugs or biologics, presentation of immune-regulatory ligands, or co-transplantation of accessory cells within a 3D scaffold, that is intentionally porous to vessels (107, 120–123).

One class of systems utilizes drug-eluting macroporous scaffolds at extrahepatic sites. Jiang et al. Incorporated dexamethasone into polydimethylsiloxane-based macroporous scaffolds designed for subcutaneous islet transplantation. Local dexamethasone releases accelerated islet engraftment and improved early glycemic control by skewing host macrophages towards an anti-inflammatory M2 phenotype, while the open scaffold architecture supported revascularization (120). Frei et al. utilized similar polydimethylsiloxane scaffolds to locally deliver fingolimod (FTY720). Although sustained drug delivery allowed for local immunomodulation, high levels of local fingolimod affected islet viability, underscoring the importance of precise control of local drug dose to preserve both vascular health and β-cell functionality (120).

More recently, Paez-Mayorga et al. Introduced the NICHE device, a pre-vascularized, implantable niche with dedicated reservoirs for immunosuppressants. Allogeneic islets were loaded into the vascularized NICHE alongside locally delivered Cytotoxic T-Lymphocyte-Associated Protein 4 (CTLA-4)-Ig and anti-lymphocyte serum in immunocompetent diabetic rats. This strategy achieved diabetes reversal for >150 days without systemic immunosuppression. Further histological analysis confirmed robust revascularization of the graft. Early nonhuman primate (NHP) work demonstrated device biocompatibility and short-term allogeneic islet engraftment (107). This design couples enhanced vascularization using a pre-formed vascular network with localized pharmacologic immune protection.

Additionally, immunomodulatory scaffolds also have the capacity to present biochemical cues that indirectly favor vascularization. Wang et al. engineered mesenchymal stromal cells to express Programmed Death Ligand-1 (PDL-1) and CTLA-4-Ig and co-transplanted them with islets. These accessory cells created a localized immunosuppressive milieu, reducing effector *T*-cell infiltration, increasing regulatory *T*-cells within the graft, and prolonging allogeneic islet survival in mice without systemic immunosuppression. Furthermore, the absence of a physical barrier allowed for normal revascularization of the islets (122).

Complementing scaffold approaches, genome editing can produce hypo-immune cells that work with local immunosuppression to minimize transplant recognition and rejection. In one work, researchers dissociated primary human islets to single cells and used CRISPR to knock-out B2M and CIITA, so the cells no longer express HLA class I or II. They then overexpressed CD47 and reaggregated the cells into pseudoislets. These synthetic pseudoislets sustained normal insulin production, resisted both *T*-cell and innate effector assaults, engrafted, and restored blood-glucose control in allogeneic and autoimmune humanized mice models. The grafts could be removed, if necessary, with an anti-CD47 antibody as a safety switch (124). The similar principle was applied to pluripotent stem cells: CD47 overexpression paired with MHC class I and II inactivation produced hypoimmunogenic iPSCs and differentiated derivatives (endothelial cells, smooth muscle cells, cardiomyocytes). Those derivatives persisted over term in fully allogeneic recipients without systemic immunosuppression. This research defines a feasible technique for linking inherent immune evasion with scaffold-delivered tolerogenic signals. They also indicate that complementary surface characteristics, such as nano-thin immunomodulatory coatings, might be applied to scaffolds to further influence local immune signals.

Furthermore, nano-thin immunomodulatory coatings can be integrated with scaffolds to combine direct vascularization with local immune signaling. Barra et al. conjugated CTLA4-Ig to antioxidant poly(N-vinylpyrrolidone)/tannic acid nanolayers on islet surfaces. The resultant localized checkpoint blockade led to suppression of macrophage STAT1 signaling, increase in regulatory and anergic T-cell populations, and significantly prolonged allograft survival in mouse models (123). When such coatings are used in conjunction with vascularized scaffolds, they offer chemical, rather than physical, immune protection while preserving the intimate contact between islets and host capillaries.

## 4 Engineering endocrine cell types beyond β-cells

The pancreas is a complex structure with endocrine and exocrine tissue. The pancreas is predominantly (∼98%) of exocrine tissue and only ∼1%–2% of endocrine tissue (125). While the endocrine pancreas is responsible for the maintenance of blood glucose homeostasis, the exocrine pancreas is responsible for secretion of pancreatic enzymes for digestion. Consequently, there are a multitude of endocrine cells that can be found within the native pancreatic tissue. β-cells are insulin producing cells that make up approximately 40%–60% of the endocrine pancreatic niche in humans (126). As such, a large amount of research has been dedicated to engineering transplantable pancreatic β-cell constructs (57, 127–130). However, β-cell bioengineering alone does not replicate the multitude of functions of the pancreas. In this section, we critically examine the current landscape in bioengineering of alpha (α)-, delta (δ)-, pancreatic polypeptide (PP)-, and exocrine cells of the pancreas (Table 3).

**Table 3:** Endocrine and exocrine cell subtypes, function, and current engineering strategies.

Cell Type	Cell Function	Strategy	References
Alpha (α)-cells	Secretes glucagon, endocrine hormone responsible for increasing blood glucose levels.	Development of α cells from iPSCs; PSC differentiation via pre-α intermediates and with controlled expression of NKX6.1.	(136, 137, 141, 142)
Delta (δ)-cells	Secretes somatostatin, hormone that inhibits glucagon and insulin to maintain glucose homeostasis.	Differentiation from PSCs into δ-cells FGF receptor-1 signaling pathway using FGF-2 and -7; somatostatin analogues tested clinically to reduce glucagon secretion and improve glycemic control.	(100, 101)
Pancreatic polypeptide (PP) cells	Secretes pancreatic polypeptide responsible for exocrine secretion and GI motility.	Limited studies available on bioengineering strategies for isolated PP cell differentiation.	(143–145)
Exocrine cells (Acinar cells)	Secretes digestive enzymes such as amylases, lipases, and proteases for digestive function.	Exocrine pancreas regeneration through pancreas derived MSCs that express islet specific and mature exocrine markers; trans-differentiation of exocrine pancreas cells into β-cells with exposure to various growth factors and transcription factors.	(146–150)
Exocrine cells (Ductal cells)	Secretes bicarbonate to neutralize acidic chyme from the stomach as it enters the duodenum.		

In addition to secretion of glucagon, alpha (α)-cells play a significant role in islet vascular development. Wieland and colleagues developed pseudo-islets composed of α-cells, β-cells, and ECs and showed that following self-assembly, ECs were found in proximity to α-cells. Additionally, with the increase in size of the pseudo-islet there was also an increase in presence of α-cells and ECs (131). Furthermore, this also holds true during development. At 19–21-week gestation, two separate cell clusters form, one composed of β-cells and the second composed of α- and δ-cells. These clusters merge to form the islet of Langerhans. During this time, ECs are preferentially found in clusters with α-cells (132). In turn, ECs are involved in the development of islet vascularization, providing ECM components such as type IV collagen, laminins, and connective tissue needed for endocrine cell development and function (133–135). There are several studies that have demonstrated development of α-cells from progenitor cells. Peterson et al. developed stem cell-derived human pancreatic α-cells from pluripotent stem cells (PSCs) via a pre-α cell intermediate. These cells demonstrate a structure similar to α-cells and express and secrete glucagon that increased glucose levels upon transplantation into mice (136). Yabe et al. also demonstrated that human PSCs can be differentiated into α-cells by controlling the expression of NKX6.1 (137).

Delta (δ)-cells secrete somatostatin, an autocrine and paracrine hormone that inhibits the secretory cells (α- and β-cells). Abnormality in δ-cell expression has been implicated in the development of T2D and progression of the disease. Previous studies have shown that β-cell: δ-cell ratio is lower in young adults with diabetes and is unchanged in healthy adults, indicating that higher number of δ-cells (138). Other studies have shown that infusion of somatostatin or its analogues in diabetic patients resulted in improved glycemic control secondary to reduction of glucagon secretion (139, 140). Therefore, δ-cells may play a pivotal role in development of therapeutics for diabetes. Multiple studies showed that FGF7 promotes endoderm differentiation, and FGF2 directs progenitor cells toward δ-cell signaling through FGF receptor-1 signaling. They were successfully able to differentiate hPSCs into δ-cells that secreted somatostatin and contributed to balanced paracrine regulation (141, 142).

PP cells secrete pancreatic polypeptide, which regulates exocrine secretion and gastrointestinal motility. They make up less than 5% of the islet cell population (143–145). Due to their scarcity, there have been limited studies evaluating PP cell potential in regenerative medicine.

Exocrine pancreas is composed of acinar cells and ductal cells. The acinar cells are the main secretory units of exocrine pancreas. Acinar cells secrete digestive enzymes, while the ductal cells secrete bicarbonate into the duodenum (125). There is a complex interplay between the endocrine and exocrine pancreas, which may have implications in management of multiple pancreatic diseases including diabetes. Kou et al. demonstrated that pancreas derived MSCs express islet specific markers and mature exocrine markers such as amylase, cytokeratin and acinar and ductal cell differentiation markers (NGN3, PTF1a and HNF1b). Additionally, they successfully demonstrated that pancreatic MSCs regenerated exocrine pancreas improved blood glucose levels in streptozotocin induced diabetic mice (146). Exocrine pancreas cells have plasticity and the ability to transdifferentiate into other cells. Several studies have shown that acinar cells can be differentiated into β-cells in the presence of various growth factors and transcription factors. They can secrete insulin; however, their secretory capacity is low compared to native β-cells (147–150).

## 5 Translational progress

Over the last two decades, the advances in vascular biology, biomaterials, and stem-cell engineering have propelled pancreatic tissue engineering from proof-of-concept studies towards clinically relevant therapies. Translational progress has been driven by rigorous validation in animal models (102, 106, 151–156) and the parallel development of bioartificial pancreas devices, together defining the translational progress towards early human application. This section will address key milestones in the translational progression of vascularized pancreatic tissue engineering, encompassing validation in animal models and the evolution of bioartificial pancreas devices intended for clinical deployment.

### 5.1 Animal models

Preclinical animal models have been crucial for demonstrating the translational potential of vascularized pancreatic tissue engineering in diabetes treatment. Most early research used streptozotocin-induced diabetic mouse and rat models, which offer a rigorous platform to evaluate graft survival and glucose regulation. These models showed that pre-vascularized extrahepatic sites, such as the subcutaneous space and perivisceral niches, reliably support long-term normoglycemia when loaded with sufficient endocrine tissue. Tissue-engineered chambers and angiogenic biomaterial–conditioned subcutaneous tracks achieved sustained diabetes reversal for over 100 days in rodents, even with minimal islet mass (102). These findings indicate that poorly perfused anatomical sites can be transformed into functional endocrine environments through controlled vascular priming. Such effects were associated with stable glucose-responsive insulin secretion and significantly improved graft survival. Forster and colleagues established a pre-vascularized chamber model in mice, which was later expanded by Pepper and Vlahos to demonstrate scalable, device-free angiogenic subcutaneous sites in both mice and rats (106).

Biomaterial-based strategies have further refined vascular and structural control of engineered pancreatic grafts in rodent and large-animal models. In diabetic mice, macroporous polycaprolactone scaffolds accelerated the return to normoglycemia and increased vascular density compared to rapidly degrading polymers, while similar constructs maintained mechanical stability and endocrine architecture in pig models, demonstrating scalability beyond rodents (151). Collagen- and fibrin-based matrices likewise improved subcutaneous islet engraftment efficiency and reduced early hypoxic graft loss in diabetic rat models (152). Collectively, these studies established that scaffold porosity, degradation kinetics, and mechanical integrity strongly influence vascular patency and long-term endocrine function across species.

At the organ level, decellularized pancreas models have enabled reconstruction of perfusable vascularized pancreatic tissue. Perfusion-decellularized rat and porcine pancreata preserve native ductal and vascular architecture and support endocrine differentiation following recellularization (153). In diabetic rat models, re-cellularized pancreatic matrices restore glycemic control and insulin production. Importantly, non-pancreatic scaffolds have also been repurposed for endocrine reconstruction. Citro et al. used decellularized lung scaffolds reseeded with ECs and neonatal islets to generate a perfusable vascularized endocrine organ that restored normoglycemia in diabetic mice, demonstrating that intact hierarchical vasculature rather than pancreas-specific anatomy alone is sufficient to sustain endocrine tissue function (154). Recent advances in 3D bioprinting and organoid-based pancreatic tissue engineering have introduced precise spatial control of endocrine and vascular architecture *in vivo*. In diabetic mouse models, bioprinted pancreatic ECM–based constructs containing perfusable vascular channels rapidly inosculated with host vessels, preserved islet architecture, and improved glucose-responsive insulin secretion compared with non-specific matrices (92). In parallel, self-organizing pancreatic organ buds composed of endocrine, endothelial, and mesenchymal cells rapidly connected to host circulation and sustained coordinated hormone secretion following transplantation, demonstrating that multicellular pancreatic micro-tissues can self-assemble into functional vascularized units in adult mice (155). The integration of PSC–derived pancreatic tissue with vascular engineering represents one of the most advanced directions in animal-model translation. Campo and colleagues generated fully hPSC–derived vascularized pancreatic micro-organs that became perfusable *in vitro* and rapidly corrected hyperglycemia after implantation into diabetic immunodeficient mice (44). These constructs displayed coordinated insulin, glucagon, and somatostatin secretion, moving beyond isolated β-cell replacement toward reconstruction of multicellular pancreatic endocrine tissue with physiological vascular coupling.

To address clinical scalability, vascularized pancreatic tissue engineering strategies have been extended into large-animal and NHP models, which better reflect human anatomy, hemodynamics, and immune complexity. The omentum has emerged as a particularly effective implantation site due to its intrinsic vascularity. In diabetic NHPs, bioengineered omental platforms using plasma–thrombin matrices enabled rapid and sustained insulin independence using allogeneic islets from a single donor per recipient, with glycemic normalization occurring within one week (156). Earlier omental scaffold studies in primates similarly demonstrated long-term glucose control. Additional NHP platforms incorporating bioengineered artificial interstitium systems with localized immune modulation have enabled prolonged xenogeneic islet survival without systemic immunosuppression, directly addressing two major translational barriers which are hypoxia and immune rejection (157). Overall, animal-model studies have demonstrated that vascularized pancreatic tissue constructs ranging from pre-vascularized endocrine niches and scaffold-based systems to decellularized organ matrices, bioprinted platforms, and fully human stem cell-derived pancreatic micro-organs can reproducibly restore endocrine function *in vivo*.

### 5.2 Bioartificial pancreas devices

Bioartificial pancreas systems aim to restore endogenous insulin secretion by combining living insulin-producing cells with mechanical or biomaterial-based immunoprotective platforms. These devices are designed to physically shield transplanted β-cells from host immune attack while maintaining sufficient diffusion of oxygen, glucose, nutrients, and insulin. Unlike conventional islet transplantation, bioartificial systems seek to eliminate or reduce the need for systemic immunosuppression while enabling long-term graft survival and physiologic glucose responsiveness (158–160).

Broadly, these platforms fall into two categories: (1) macro-encapsulation devices, which house large numbers of cells within retrievable devices implanted subcutaneously or intraperitoneally, and (2) pre-vascularized pouch systems, which create a biologically permissive niche for later cell implantation. Leading clinical-stage examples include ViaCyte, Sernova Cell Pouch, and TheraCyte/Boettler-based encapsulation devices. The aim of this section is to briefly introduce commercially available bioartificial pancreas devices. Pertinent clinical trials will be discussed in a later section (See Section 5.3).

#### 5.2.1 Viacyte

ViaCyte has developed a series of stem cell-derived pancreatic progenitor cell devices utilizing a macro-encapsulation strategy designed for subcutaneous implantation. The first-generation of PEC-Encap (VC-01) system encapsulated human ESC-derived pancreatic progenitors within a semi-permeable membrane (Encaptra®), allowing for immunoisolation while maintaining exchange of key nutrients and insulin secretion.

ViaCyte later partnered with Clustered Regulatory Interspaced Short Palindromic Repeats (CRISPR) Therapeutics to develop gene-edited immune-evasive cell platforms (VCTX210) aimed at eliminating the need for systemic immunosuppression.

#### 5.2.2 TheraCyte/Boettler devices

TheraCyte is another macro-encapsulation device consisting of a bilaminar Polytetrafluoroethylene membrane that allows for diffusion of oxygen and nutrients while blocking immune cells. Pre-clinical studies demonstrated short-term protection of xenogeneic islets from rejection (160). However, long-term graft survival was severely limited by fibrotic capsule formation and hypoxia driven β-cell loss.

The Boettler device platform introduced refinements in polymer chemistry and membrane permeability to improve diffusion kinetics and mechanical stability. Although animal studies demonstrated glycemic correction, translation to durable human application has been hindered by foreign body response, insufficient oxygen delivery, and necrotic graft cores; a recurring failure mode across diffusion-dependent encapsulation platforms (158, 159).

#### 5.2.3 Sernova cell pouch system

The Sernova Cell Pouch System employs a different strategy based on pre-vascularized tissue engineering. The device is initially implanted subcutaneously as an empty scaffold to induce host-driven neovascularization and formation of highly vascularized stromal pouch prior to cell-delivery. Following vascularization, donor islets or stem cell-derived endocrine cells are secondarily implanted into the pre-vascularized device.

Phase I/II clinical trials in patients with brittle T1D demonstrated successful vascularized engraftment, sustained C-peptide production, improved glycemic control, and protection from severe hypoglycemia, even with relatively modest islet numbers (161, 162). More importantly, histologic and imaging analyses confirmed the formation of dense, functional microvascular networks within the pouch, directly supporting β-cell survival and function.

Unlike fully immunoisolated macro-encapsulation devices, Sernova still requires systemic immunosuppression. However, its success highlights that pre-established vascular integration strategy dramatically improves graft survival and function compared with diffusion-limited macro-encapsulation devices.

### 5.3 Clinical trials

#### 5.3.1 Omental bioHub (Diabetes Research Institute, USA)

The BioHub is a tissue-engineered platform developed at the Diabetes Research Institute that repurposes the omentum as a vascularized implantation site for pancreatic islets. In an ongoing clinical study of allogeneic islet transplantation onto the omentum (CI: NCT02213003), a woman with long-standing T1D and hypoglycemia unawareness received ∼602,000 islet equivalents from a single donor embedded in a plasma–thrombin biodegradable scaffold and implanted laparoscopically onto the omentum under systemic immunosuppression. Insulin was discontinued 17 days after transplantation, and at 12 months the patient maintained euglycemia with an HbA1c of ∼6.0%, sustained endogenous C-peptide secretion, and complete elimination of severe hypoglycemia. Although insulin secretion declined modestly over time, the patient remained insulin-independent with stable glycemic control, establishing the omentum as a functional, vascularized extrahepatic site for bioengineered islet transplantation in humans (163).

#### 5.3.2 *β*air device (Beta-O2 Technologies, Israel)

The *β*Air system is a macro-encapsulation device designed to maintain islet viability through active oxygen supplementation while physically isolating the graft from host immune cells. Islets are housed within a sealed chamber separated from host tissue by diffusion membranes, and oxygen is supplied through a refillable subcutaneous port to prevent hypoxic injury. In a Phase I clinical study (CI: NCT02064309), the device was implanted subcutaneously in individuals with long-standing T1D. Each subject received 1–2 devices containing 155,000–180,000 allogeneic human islet equivalents and was monitored for 3–6 months before device retrieval. The device proved safe and effectively prevented immune rejection and transplanted β-cells remained viable. However, only minimal circulating C-peptide was detected with no significant metabolic benefit, alongside fibrosis, immune cell accumulation, blunted glucose-stimulated insulin secretion, and amyloid formation, indicating limited functional efficacy despite cell survival. The *β*Air platform is currently in phase 2 clinical evaluation (159).

#### 5.3.3 PEC-Encap (VC-01, ViaCyte, USA)

PEC-Encap-derived grafts have pushed the field further toward scalable, engineered endocrine organs. Early trials of human ESC-derived pancreatic endoderm in subcutaneous encapsulation devices (VC-01/PEC-Encap) demonstrated engraftment and insulin expression in humans but only modest C-peptide and limited glycemic benefit, largely because peri-device fibrosis impeded vascular ingrowth, and the program was discontinued (CI: NCT04678557). In a follow-on approach, non-immunoprotective macro-encapsulation devices that allow direct vascularization but require systemic immunosuppression (VC-02/PEC-Direct) yielded more robust endocrine function: in a phase 1/2 cohort, 63% of explanted devices showed engraftment, and 6/17 patients developed new fasting and glucose-responsive mixed-meal C-peptide secretion, though none became insulin-independent. (CI: NCT03163511) (164, 165).

#### 5.3.4 VX-880 (Zimislecel) from Vertex pharmaceuticals

VX-880 (Zimislecel) is a suspension of fully differentiated allogeneic stem cell–derived pancreatic islet cells developed by Vertex Pharmaceuticals. Unlike progenitor-based approaches, VX-880 delivers mature insulin-producing cells directly into the portal circulation, allowing immediate integration with the native hepatic vascular network. This approach requires standard systemic immunosuppression but eliminates the need for macro-devices. In the ongoing Phase I/II forward trial (CI: NCT04786262), VX-880 produced the most striking clinical outcomes in stem cell–based diabetes therapy to date. All fully dosed participants developed durable endogenous C-peptide secretion, elimination of severe hypoglycemia, and marked improvements in time-in-range and HbA1c. Importantly, approximately 80% of fully dosed patients achieved insulin independence at 1 year, prompting advancement of VX-880 into a pivotal Phase I/II/III clinical program (166). A sister product VX-264, is currently in Phase II. Collectively, these trials show that clinically effective bioengineered pancreatic replacement is already achievable, but long-term success still hinges on balancing robust vascular integration with durable immune protection and acceptable immunosuppression.

Early clinical exploration of stem-cell derived islet replacement has taken a notable step with development of an alternative site for islet cell implantation. Conventionally, pancreatic islet cells are delivered through the portal vein and transplanted into the liver where they are allowed to revascularize. However, this method is limited by graft loss secondary to IBMIR. Thus, alternative implantations sites (e.g., omentum, subcutaneous space, intramuscular, marrow, anterior rectus sheath) have been proposed to prevent long-term graft attrition. Wang et al. Demonstrated feasibility of a self-contained extraperitoneal transplant site using the anterior rectus sheath at the abdomen. Furthermore, they found that implantation in the anterior rectus sheath led to superior CiPSC-islet survival and maturation.

## 6 Key challenges in clinical translation

Although clinical trials now clearly demonstrate that vascularized pancreatic tissue engineering can restore meaningful endocrine function in patients with diabetes, several critical challenges continue to limit broad clinical implementation. Foremost among these is immune rejection, as virtually all strategies that achieve strong metabolic outcomes currently depend on lifelong systemic immunosuppression. While effective for graft survival, chronic immunosuppression exposes patients to risks of infection, malignancy, renal toxicity, and metabolic complications, making it an imperfect long-term solution for a disease that is otherwise manageable with exogenous insulin. Encapsulation-based approaches were developed to avoid these risks; however, clinical experience has shown that immune isolation often comes at the cost of inadequate vascular integration, leading to hypoxia, diffusion-limited nutrient delivery, and insufficient insulin output. This persistent tension between robust vascular access and durable immune protection remains one of the most fundamental unresolved problems in the field. A related concern is long-term graft durability. Even in the most successful trials, declines in endogenous insulin secretion have been observed over time, and truly long-term human follow-up beyond five to ten years is still lacking. It remains unclear whether engineered pancreatic grafts will maintain stable function over decades or whether periodic retreatment will be required. This uncertainty complicates both patient selection and long-term therapeutic planning. At the same time, the scalability and manufacturing consistency of these therapies pose substantial barriers to widespread clinical use. The production of stem cell-derived pancreatic tissue under good manufacturing practice conditions is technically demanding, resource-intensive, and costly, raising important questions about affordability, accessibility, and health system integration.

Finally, surgical complexity and device management continue to impose practical limitations. Some platforms require invasive implantation procedures or rely on anatomical sites that are difficult to access for monitoring or retrieval, complicating long-term follow-up and graft replacement. Device-based systems may also face issues related to mechanical failure, fibrosis, or loss of function over time. Together, these biological, engineering, and logistical challenges underscore that while vascularized pancreatic tissue replacement has moved from experimental concept to clinical reality, its routine clinical use will ultimately depend on achieving immune-compatible vascularization, long-term graft stability, scalable manufacturing, and safe, practical surgical delivery

## 7 Ethical and regulatory considerations

The convergence of stem cell biology, genome edition, biomaterials, and implantable devices in vascularized pancreatic tissue engineering raises a distinct set of ethical and regulatory challenges. At the most basic level, β-cell replacement therapies are being developed for a chronic, otherwise manageable disease, which heightens the threshold for acceptable risk relative to oncology or terminal indications. Thus, invasive implantation procedures, lifelong or intermittent immunosuppression, and uncertain long-term graft behavior must be weighed against the risks or burdens of intensive insulin therapy. Transparent communication of these trade-offs, including realistic expectations about durability and retreatment, is essential to ethically sound informed consent.

PSC-derived products raise additional concerns. Human ESC-derived β-cells remain ethically contentious in some jurisdictions due to embryo use, leading to divergent national regulations and funding restrictions. iPSCs mitigate many of these concerns but introduce tumorigenicity risks if residual undifferentiated cells or proliferative off-target populations persist within the final product (69, 167, 168). Regulatory agencies now expect rigorous biodistribution and tumorigenicity testing for pluripotent stem cell-derived therapies. Furthermore, they require long-term *in vivo* follow-up and sensitive assays to detect rare proliferative clones (119–121). These requirements influence trial design, patient monitoring, and manufacturing stringency.

Gene-edited “immune-evasive” β-cells introduce further ethical and oversight questions. Programs such as VCTX210, which combine CRISPR-mediated editing of HLA and immune checkpoints with stem cell-derived pancreatic progenitors, are explicitly designed to avoid systemic immunosuppression (169). While the CRISPR-mediated edits are somatic rather than germline, they result in permanent genomic modifications. Thus, these are not without theoretical risks of off-target mutations, altered immunosurveillance, or unexpected fitness advantages. Long-term registries and transparent data sharing will be necessary to assess whether immune-evasive cell lines behave predictably long-term, or if they contribute to secondary malignancies or opportunistic infections. Equitable access is a concern; gene-edited and autologous iPSC products are likely to be costly, raising the risk that transformative therapies will be restricted to a small subset of patients in high-resource settings.

Regulation of bioartificial pancreas therapies is complicated by their hybrid nature as cell-device combination products. In the United States, allogeneic islet products are regulated as biologic drugs requiring a Biologics License Application with potency, purity, and manufacturing standards (170, 171). Simultaneously, encapsulation devices, macro-chambers, and pre-vascularized pouches fall under medical device or combination-product regulation, covered by separate pathways and post-market obligations (172, 173). This fragmented framework can slow clinical translation and create uncertainty over classification. In the United States, professional societies like the American Society of Transplant Surgeons continue to advocate for islet cell allotransplantation to be regulated as an organ transplant rather than a drug (174, 175). In contrast, Canada and Europe recognize ICT as a standard of care in select patients (176, 177). Ethically, this divergence raises questions about global justice in which patients in some regions have access to established islet therapies under organ transplant rules, while others must wait for drug-like approval of similar or related products.

Finally, robust governance is needed around trial design, risk-benefit balance, and patient selection. Early-phase studies of encapsulation devices and stem cell-derived products have generally enrolled individuals with severe hypoglycemia and impaired awareness. With more evidence for strong glycemic control from islet products, there will be pressure to expand indications to broader T1D populations. Regulators and investigators will need to define clear thresholds for safety, durability, and quality-of-life improvement prior to offering these interventions to patients who can achieve near-normal glycemic control with advanced insulin technologies. Furthermore, it is imperative that commercial incentives do not outpace evidence-based patient selection.

## 8 Future perspectives

### 8.1 Nanoenabled oxygen delivery

Nanoenabled oxygen delivery is a rapidly evolving area of biomedical engineering and involves the use of nanotechnology-based systems [e.g., catalytic oxygen-generating nanocarrier, perfluorocarbon (PFC) oxygen carriers, or microfluidic oxygenators] to transport or generate oxygen in targeted tissues. Particularly, this technology has shown efficacy in reversal of tumor hypoxia, a major contributor to radiation treatment resistance, in preclinical models. Studies on hypoxic tumor cells using Oil-based and synthetic PFC droplets have demonstrated appropriate accumulation of PFC agents in solid tumors and subsequent reoxygenation of tumor cells without inhaled supplemental oxygen in mice (178–180). This is a rapidly evolving area in biomedical engineering and has emerged as a promising strategy to overcome hypoxia in tumors, chronic wounds, and other native tissues following ischemic injury (e.g., heart, brain, lungs). A major limitation to islet or engineered pancreatic tissues engraftment is acute hypoxic stress secondary to diffusion limitations, poor vascularization following implantation (38). While current strategies (e.g., pre-vascularization, angiogenic factor delivery) help, oxygen supply remains a critical bottleneck. To this date, the role of nanoenabled oxygen delivery in overcoming hypoxic stress in islet grafts or engineered pancreatic constructs has not been examined. However, this technology may be useful in providing immediate oxygenation to transplanted cells/tissue post-implantation, which may lead to subsequent islet viability during the pre-vascularization period.

## 9 Conclusion

Pancreatic tissue engineering has progressed from conceptual promise to tangible clinical reality. Whole-organ pancreas and ICT established that restoration of endogenous insulin secretion can normalize glycemic control and eliminate hypoglycemia unawareness. However, both approaches remain constrained by donor scarcity, operative morbidity, and dependence on systemic immunosuppression. These limitations have driven the development of vascularized pancreatic constructs as an alternative for β-cell replacement strategies.

Across various platforms, vascularization remains the critical bottleneck of durable bioengineered pancreatic construct. Continued progress will require not only sustained technological innovation but also careful ethical oversight and regulatory adaptation. If these challenges can be met, vascularized pancreatic tissue engineering may move beyond experimental rescue of brittle diabetes towards a broadly accessible, bioengineered cure.

## References

[1] DeFronzo RA Ferrannini E Groop L Henry R Hernan W Holst J. Type 2 diabetes mellitus. Nat Rev Dis Primers. (2015) 1(1):15019. 10.1038/nrdp.2015.19 27189025

[2] Atkinson MA Eisenbarth GS Michels AW. Type 1 diabetes. Lancet. (2014) 383:69–82. 10.1016/S0140-6736(13)60591-7 23890997 PMC4380133

[3] Group TDC and CTR. The effect of intensive treatment of diabetes on the development and progression of long-term complications in insulin-dependent diabetes Mellitus. N Engl J Med. (1993) 329(14):977–86. 10.1056/NEJM199309303291401 8366922

[4] Rickels MR Paul Robertson R. Pancreatic islet transplantation in humans: recent progress and future directions. Endocr Rev. (2019) 40(2):631–68. 10.1210/ER.2018-00154 30541144 PMC6424003

[5] Shapiro AMJ Lakey JRT Ryan EA Korbut G Toth E Warnock G. Islet transplantation in seven patients with type 1 diabetes Mellitus using a glucocorticoid-free immunosuppressive regimen. N Engl J Med. (2000) 343(4):230–8. 10.1056/NEJM200007273430401 10911004

[6] Abuazzam F Patel H Vutam M Woodside K El Chediak A Parsons R. Outcomes and predictors of pancreas graft survival in simultaneous pancreas-kidney transplant recipients: a contemporary analysis of OPTN database. Am J Transplant. (2025) 25(8):S197. 10.1016/j.ajt.2025.07.435

[7] Kandaswamy R Stock PG Gustafson SK Skeans MA Curry MA Prentice MA. OPTN/SRTR 2015 annual data report: pancreas. Am J Transplant. (2017) 17:117–73. 10.1111/AJT.14125 28052606

[8] Bosco D Armanet M Morel P Niclauss N Sgroi A Muller Y. Unique arrangement of *α*- and *β*-cells in human islets of langerhans. Diabetes. (2010) 59(5):1202–10. 10.2337/DB09-1177 20185817 PMC2857900

[9] Brereton MF Vergari E Zhang Q Clark A. Alpha-, Delta- and PP-cells: are they the architectural cornerstones of islet structure and co-ordination? J Histochem Cytochem. (2015) 63(8):575–91. 10.1369/0022155415583535 26216135 PMC4530398

[10] Jansson L Barbu A Bodin B Drott C Johan E Daniel G. Pancreatic islet blood flow and its measurement. Ups J Med Sci. (2016) 121(2):81–95. 10.3109/03009734.2016.1164769 27124642 PMC4900068

[11] Suszynski TM Avgoustiniatos ES Papas KK. Oxygenation of the intraportally transplanted pancreatic islet. J Diabetes Res. (2016) 2016(1):7625947. 10.1155/2016/7625947 27872862 PMC5107248

[12] Pignatelli C Campo F Neroni A Piemonti L Citro A. Bioengineering the vascularized endocrine pancreas: a fine-tuned interplay between vascularization, extracellular-matrix-based scaffold architecture, and insulin-producing cells. Transpl Int. (2022) 35:10555. 10.3389/TI.2022.10555 36090775 PMC9452644

[13] Gruessner AC Gruessner RWG. Long-term outcome after pancreas transplantation: a registry analysis. Curr Opin Organ Transplant. (2016) 21(4):377–85. 10.1097/MOT.0000000000000331 27258580

[14] Gruessner AC. A decade of pancreas transplantation—a registry report. Uro. (2023) 3(2):132–50. 10.3390/URO3020015

[15] Nagendra L Fernandez CJ Pappachan JM. Simultaneous pancreas-kidney transplantation for end-stage renal failure in type 1 diabetes mellitus: current perspectives. World J Transplant. (2023) 13(5):208–20. 10.5500/wjt.v13.i5.208 37746036 PMC10514751

[16] Kaye AD Shah SS Johnson CD DeWitt AS Thomassen AS Daniel CP. Tacrolimus- and mycophenolate-mediated toxicity: clinical considerations and options in management of post-transplant patients. Curr Issues Mol Biol. (2024) 47(1):2. 10.3390/CIMB47010002 39852117 PMC11763814

[17] David A Frampas E Douane F Perret C Leute F Cantarovich D. Management of vascular and nonvascular complications following pancreas transplantation with interventional radiology. Diagn Interv Imaging. (2020) 101(10):629–38. 10.1016/J.DIII.2020.02.002 32089482

[18] Axelrod DA Sung RS Meyer KH Wolfe RA Kaufman DB. Systematic evaluation of pancreas allograft quality, outcomes and geographic variation in utilization. Am J Transplant. (2010) 10(4):837–45. 10.1111/J.1600-6143.2009.02996.X 20121753

[19] Saidi RF Kenari SKH. Challenges of organ shortage for transplantation: solutions and opportunities. Int J Organ Transplant Med. (2014) 5(3):87–96. 25184029 PMC4149736

[20] Hering BJ Clarke WR Bridges ND Eggerman TL Alejandro R Bellin MD. Phase 3 trial of transplantation of human islets in type 1 diabetes complicated by severe hypoglycemia. Diabetes Care. (2016) 39(7):1230–40. 10.2337/DC15-1988 27208344 PMC5317236

[21] Vantyghem MC Defrance F Quintin D Leroy C Raverdi V Prévost G. Treating diabetes with islet transplantation: lessons from the past decade in lille. Diabetes Metab. (2014) 40(2):108–19. 10.1016/j.diabet.2013.10.003 24507950

[22] Perrier Q Jambon-Barbara C Kessler L Villard O Buron F Guerci B. Impact of islet transplantation on diabetes complications and mortality in patients living with type 1 diabetes. Diabetes Care. (2025) 48(6):1007–15. 10.2337/DC25-0059 40245107 PMC12094206

[23] Brennan DC Kopetskie HA Sayre PH Alejandro R Cagliero E Shapiro AMJ. Long-Term follow-up of the Edmonton protocol of islet transplantation in the United States. Am J Transplant. (2016) 16(2):509–17. 10.1111/AJT.13458 26433206

[24] Biarnés M Montolio M Nacher V Raurell M Soler J Montanya E. *β*-Cell death and mass in syngeneically transplanted islets exposed to short- and long-term hyperglycemia. Diabetes. (2002) 51(1):66–72. 10.2337/diabetes.51.1.66 11756324

[25] Bennet W Sundberg B Lundgren T Tibell A Groth C Richards A. Damage to porcine islets of langerhans after exposure to human blood *in vitro*, or after intraportal transplantation to cynomologus monkeys: protective effects of sCR1 and heparin. Transplantation. (2000) 69(5):711–9. 10.1097/00007890-200003150-00007 10755515

[26] Nilsson B Ekdahl KN Korsgren O. Control of instant blood-mediated inflammatory reaction to improve islets of langerhans engraftment. Curr Opin Organ Transplant. (2011) 16(6):620–6. 10.1097/mot.0b013e32834c2393 21971510

[27] Carlsson PO Palm F Andersson A Liss P. Markedly decreased oxygen tension in transplanted rat pancreatic islets irrespective of the implantation site. Diabetes. (2001) 50(3):489–95. 10.2337/diabetes.50.3.489 11246867

[28] Vendrame F Pileggi A Laughlin E Allende G Martin-Pagola A Damaris Molano R. Recurrence of type 1 diabetes after simultaneous pancreas-kidney transplantation, despite immunosuppression, is associated with autoantibodies and pathogenic autoreactive CD4T-cells. Diabetes. (2010) 59(4):947–57. 10.2337/db09-0498 20086230 PMC2844842

[29] Burk GW Vendrame F Pileggi A Ciancio G Reijonen H Pugliese A. Recurrence of autoimmunity following pancreas transplantation. Curr Diab Rep. (2011) 11(5):413–9. 10.1007/s11892-011-0206-y 21660419 PMC4018301

[30] Roep BO Stobbe I Duinkerken G Van Rood JJ Lernmark A Keymeulen B. Auto- and alloimmune reactivity to human islet allografts transplanted into type 1 diabetic patients. Diabetes. (1999) 48(3):484–90. 10.2337/DIABETES.48.3.484 10078547

[31] Braghi S Bonifacio E Secchi A Di Carlo V Pozza G Bosi E. Modulation of humoral islet autoimmunity by pancreas allotransplantation influences allograft outcome in patients with type 1 diabetes. Diabetes. (2000) 49(2):218–24. 10.2337/diabetes.49.2.218 10868938

[32] Benichou G Takizawa PA Olson CA McMillan M Sercarz EE. Donor major histocompatibility complex (MHC) peptides are presented by recipient MHC molecules during graft rejection. J Exp Med. (1992) 175(1):305. 10.1084/jem.175.1.305 1730925 PMC2119070

[33] Coppieters KT Dotta F Amirian N Campbell PD Kay TWH Attkinson M. Demonstration of islet-autoreactive CD8T cells in insulitic lesions from recent onset and long-term type 1 diabetes patients. J Exp Med. (2012) 209(1):51–60. 10.1084/JEM.20111187 22213807 PMC3260877

[34] Long AE George G Williams CL. Persistence of islet autoantibodies after diagnosis in type 1 diabetes. Diabetic Med. (2021) 38(12):e14712. 10.1111/DME.14712 34614253

[35] Burke GW Vendrame F Pileggi A Ciancio G Reijonen H Pugliese A. Recurrence of autoimmunity following pancreas transplantation. Curr Diab Rep. (2011) 11(5):413–9. 10.1007/S11892-011-0206-Y 21660419 PMC4018301

[36] Larsen JL. Pancreas transplantation: indications and consequences. Endocr Rev. (2004) 25(6):919–46. 10.1210/ER.2002-0036 15583023

[37] Traitanon O Mathew JM La Monica G Xu L Mas V Gallon L. Differential effects of tacrolimus versus sirolimus on the proliferation, activation and differentiation of human B cells. PLoS One. (2015) 10(6):e0129658. 10.1371/journal.pone.0129658 26087255 PMC4472515

[38] Einstein SA Steyn LV Weegman BP Bradley P Suszynski TM Samanis A. Hypoxia within subcutaneously implanted macroencapsulation devices limits the viability and functionality of densely loaded islets. Front Transplant. (2023) 2:1257029. 10.3389/FRTRA.2023.1257029 38993891 PMC11235299

[39] Townsend SE Gannon M. Extracellular matrix–associated factors play critical roles in regulating pancreatic *β*-cell proliferation and survival. Endocrinology. (2019) 160(8):1885–94. 10.1210/EN.2019-00206 31271410 PMC6656423

[40] Mangani S Vetoulas M Mineschou K Spanopoulos K Vivanco M Piperigkou Z. Design and applications of extracellular matrix scaffolds in tissue engineering and regeneration. Cells. (2025) 14(14):1076. 10.3390/CELLS14141076 40710329 PMC12293650

[41] Uday Chandrika K Tripathi R Kameshwari Y Rangaraj N Mahesh Kumar J Singh S. Refunctionalization of decellularized organ scaffold of pancreas by recellularization: whole organ regeneration into functional pancreas. Tissue Eng Regen Med. (2020) 18(1):99–112. 10.1007/S13770-020-00296-Y 33098547 PMC7862468

[42] Hao L Khajouei F Rodriguez J Kim S Lee EJA. Unlocking the promise of decellularized pancreatic tissue: a novel approach to support angiogenesis in engineered tissue. Bioengineering (Basel). (2024) 11(2):183. 10.3390/bioengineering11020183 38391669 PMC10886056

[43] Sevastianov VI Ponomareva AS Baranova NV Belova AD Kirsanova LA Nikolskaya AO. A tissue-engineered construct based on a decellularized scaffold and the islets of langerhans: a streptozotocin-induced diabetic model. Life. (2024) 14(11):1505. 10.3390/LIFE14111505 39598303 PMC11595861

[44] Banerjee D Nayakawde NB Antony D Deshmukh M Ghosh S Sihlbom C. Characterization of decellularized implants for extracellular matrix integrity and immune response elicitation. SAGE J. (2022) 28(13-14):621–39. 10.1089/TEN.TEA.2021.0146 34963315

[45] Veiseh O Doloo JC Ma M Vegas AJ Hei Tam H Bader AR. Size- and shape-dependent foreign body immune response to materials implanted in rodents and non-human primates. Nat Mater. (2015) 14(6):643–51. 10.1038/NMAT4290 25985456 PMC4477281

[46] Lansberry TR Accolla RP Crouse CC Miravet IL Walsh J Molano RR. Optimizing 3D-printed Scaffold Geometry Decreases Foreign Body Response and Enhances Allogeneic Islet Transplant Outcomes. bioRxiv. Published online 2026:2026.02.02.701816-2026.02.02.701816. 10.64898/2026.02.02.701816

[47] Wang X Maxwell KG Wang K Bowers DT Flanders JA Liu W. A nanofibrous encapsulation device for safe delivery of insulin-producing cells to treat type 1 diabetes. Sci Transl Med. (2021) 13(596):eabb4601. 10.1126/scitranslmed.abb4601 34078744 PMC8563008

[48] Friedrich EE Lanier ST Niknam-Bienia S Arenas GA Rajendran D Wertheim JA. Residual sodium dodecyl sulfate in decellularized muscle matrices leads to fibroblast activation *in vitro* and foreign body response *in vivo*. J Tissue Eng Regen Med. (2018) 12(3):e1704–15. 10.1002/term.2604 29084373

[49] Diedrich AM Daneshgar A Tang P Klein O Mohr A Onwuegbuchulam OA. Proteomic analysis of decellularized mice liver and kidney extracellular matrices. J Biol Eng. (2024) 18(1):17. 10.1186/s13036-024-00413-8 38389090 PMC10885605

[50] Xu T Zhu M Guo Y Wu D Huang Y Fan X. Three-dimensional culture of mouse pancreatic islet on a liver-derived perfusion-decellularized bioscaffold for potential clinical application. J Biomater Appl. (2015) 30(4):379–87. 10.1177/0885328215587610 26006767

[51] Uygun BE Soto-Gutierrez A Yagi H Izamis ML Guzzardi MA Shulman C. Organ reengineering through development of a transplantable recellularized liver graft using decellularized liver matrix. Nat Med. (2010) 16(7):814–20. 10.1038/nm.2170 20543851 PMC2930603

[52] Ott HC Clippinger B Conrad C Schuetz C Pomerantseva I Ikonomou L. Regeneration and orthotopic transplantation of a bioartificial lung. Nat Med. (2010) 16(8):927–33. 10.1038/nm.2193 20628374

[53] Ren X Moser PT Gilpin SE Okamoto T Wu T Tapias LF. Engineering pulmonary vasculature in decellularized rat and human lungs. Nat Biotechnol. (2015) 33(10):1097–102. 10.1038/nbt.3354 26368048

[54] Gujjar S Tyagi A Sainger S Bharti P Nain V Sood P. Biocompatible human placental extracellular matrix derived hydrogels. Adv Biol. (2024) 8(1):2300349. 10.1002/adbi.202300349 37786307

[55] Asgari F Asgari HR Najafi M Eftekhari BS Vardiani M Gholipourmalek AM. Optimization of decellularized human placental macroporous scaffolds for spermatogonial stem cells homing. J Mater Sci: Mater Med. (2021) 32(5):47. 10.1007/s10856-021-06517-7 33891169 PMC8065005

[56] Bogomolova A Ermakova P Potapov A Mozherov A Tselousova J Vasilchikova E. *In Vitro* and *in vivo* testing of decellularized lung and pancreas matrices as potential islet platforms. Int J Mol Sci. (2025) 26(14):6692. 10.3390/ijms26146692 40724943 PMC12294404

[57] Pagliuca FW Millman JR Gürtler M Segal M Van Dervort A Ryu JH. Generation of functional human pancreatic *β* cells *in vitro*. Cell. (2014) 159(2):428–39. 10.1016/j.cell.2014.09.040 25303535 PMC4617632

[58] Asumda FZ Alzoubi S Padarath K John N Jones K Kolhe R. Efficient generation of induced pluripotent stem cell-derived definitive endoderm cells with growth factors and small molecules. Cells. (2025) 14(11):815. 10.3390/CELLS14110815 40497991 PMC12153844

[59] Bogacheva MS Harjumäki R Flander E Taalas A Bystriakova M Yliperttula M. Differentiation of human pluripotent stem cells into definitive endoderm cells in Various flexible three-dimensional cell culture systems: possibilities and limitations. Front Cell Dev Biol. (2021) 9:726499. 10.3389/FCELL.2021.726499 34568336 PMC8459831

[60] Memon B Abdelalim EM. Toward precision medicine with human pluripotent stem cells for diabetes. Stem Cells Transl Med. (2022) 11(7):704–14. 10.1093/STCLTM/SZAC030 35640144 PMC9299517

[61] Ilic D Ogilvie C. Pluripotent stem cells in clinical setting—new developments and overview of current Status. Stem Cells. (2022) 40(9):791–801. 10.1093/STMCLS/SXAC040 35671338 PMC9512105

[62] Tran R Moraes C Hoesli CA. Controlled clustering enhances PDX1 and NKX6.1 expression in pancreatic endoderm cells derived from pluripotent stem cells. Sci Rep. (2020) 10(1):1190. 10.1038/s41598-020-57787-0 31988329 PMC6985188

[63] McGaugh EC Nostro MC. Efficient differentiation of pluripotent stem cells to NKX6-1+ pancreatic progenitors. J Vis Exp. (2017) (121):e55265. 10.3791/55265 PMC5409288 28362406

[64] Braam MJS Zhao J Liang S Ida S Kloostra NK Iworima DG. Protocol development to further differentiate and transition stem cell-derived pancreatic progenitors from a monolayer into endocrine cells in suspension culture. Sci Rep. (2023) 13(1):1–17. 10.1038/S41598-023-35716-1 37264038 PMC10235054

[65] Arroyave F Uscátegui Y Lizcano F. From iPSCs to pancreatic *β* cells: unveiling molecular pathways and enhancements with vitamin C and retinoic acid in diabetes research. Int J Mol Sci. (2024) 25(17):9654. 10.3390/IJMS25179654 39273600 PMC11395045

[66] Li XY Zhai WJ Teng CB. Notch signaling in pancreatic development. Int J Mol Sci. (2015) 17(1):48. 10.3390/IJMS17010048 26729103 PMC4730293

[67] Braam MJS Zhao J Liang S Ida S Kloostra NK Iworima DG. Protocol development to further differentiate and transition stem cell-derived pancreatic progenitors from a monolayer into endocrine cells in suspension culture. Sci Rep. (2023) 13(1):8877. 10.1038/S41598-023-35716-1 37264038 PMC10235054

[68] Diane A Mu-U-Min RBA Al-Siddiqi HH. Epigenetic memory as crucial contributing factor in directing the differentiation of human iPSC into pancreatic *β*-cells *in vitro*. Cell Tissue Res. (2025) 399(3):267–76. 10.1007/S00441-025-03952-8 39883142 PMC11870940

[69] Pellegrini S Zamarian V Sordi V. Strategies to improve the safety of iPSC-derived *β* cells for *β* cell replacement in diabetes. Transpl Int. (2022) 35:10575. 10.3389/TI.2022.10575 36090777 PMC9448870

[70] Huan Z Luo Z Li J Yu Y Li L. Bio-Inspired pancreas with microfluidic multi-component hydrogel microfibers for exploring pancreatic exocrine and endocrine interactions. Aggregate. (2025) 12(6):e70210. 10.1002/AGT2.70210

[71] Moreno-Castellanos N Velásquez-Rincón MC Rodríguez-Sanabria AV Cuartas-Gómez E Vargas-Ceballos O. Encapsulation of beta-pancreatic cells in a hydrogel based on alginate and graphene oxide with high potential application in the diabetes treatment. J Mater Res. (2023) 38(10):2823–37. 10.1557/s43578-023-01009-6

[72] Wang S Du Y Zhang B Meng G Liu Z Liew SY. Transplantation of chemically induced pluripotent stem-cell-derived islets under abdominal anterior rectus sheath in a type 1 diabetes patient. Cell. (2024) 187(22):6152–6164.e18. 10.1016/j.cell.2024.09.004 39326417

[73] Heaton ES Hu M Liu T Hui H Tan Y Ye K. Extracellular matrix-derived peptide stimulates the generation of endocrine progenitors and islet organoids from iPSCs. J Tissue Eng. (2023):14L20417314231185858. 10.1177/20417314231185858 PMC10331343 37435573

[74] Cherubini A Rusconi F Piras R Wächtershäuser K Dossena M Barilani M. Exploring human pancreatic organoid modelling through single-cell RNA sequencing analysis. Commun Biol. (2024) 7(1):1527. 10.1038/s42003-024-07193-3 39558019 PMC11574267

[75] Nair GG Liu JS Russ HA Tran S Saxton MS Chen R. Recapitulating endocrine cell clustering in culture promotes maturation of human stem-cell-derived *β* cells. Nat Cell Biol. (2019) 21(2):263–74. 10.1038/s41556-018-0271-4 30710150 PMC6746427

[76] Rawal S Williams SJ Ramachandran K Stehno-Bittel L. Integration of mesenchymal stem cells into islet cell spheroids improves long-term viability, but not islet function. Islets. (2017) 9(5):87–98. 10.1080/19382014.2017.1341455 28662368 PMC5624285

[77] Honarpisheh M Lei Y Follenzi A Cucci A Olgasi C Berishvili E. Spheroids composed of reaggregated neonatal porcine islets and human endothelial cells accelerate development of normoglycemia in diabetic mice. Cells. (2025) 14(5):366. 10.3390/cells14050366 40072094 PMC11898817

[78] Ali A Kurome M Kessler B Kemter E Wolf E. What genetic modifications of source pigs are essential and sufficient for cell, tissue, and organ Xenotransplantation? Transpl Int. (2024) 37:13681. 10.3389/ti.2024.13681 39697899 PMC11652200

[79] Estrada JL Martens G Li P Adams A Newell KA Ford ML. Evaluation of human and non-human primate antibody binding to pig cells lacking GGTA1/CMAH/*β*4GalNT2 genes. Xenotransplantation. (2015) 22(3):194–202. 10.1111/xen.12161 25728481 PMC4464961

[80] Zhang R Wang Y Chen L Wang R Li C Li X. Reducing immunoreactivity of porcine bioprosthetic heart valves by genetically-deleting three major glycan antigens, GGTA1/*β*4GalNT2/CMAH. Acta Biomater. (2018) 72:196–205. 10.1016/j.actbio.2018.03.055 29631050

[81] Singireddy S Tully A Galindo J Ayares D Singh AK Mohiuddin MM. Genetic engineering of donor pig for the first human cardiac Xenotransplantation: combatting rejection, coagulopathy, inflammation, and excessive growth. Curr Cardiol Rep. (2023) 25(11):1649–56. 10.1007/s11886-023-01978-4 37938425 PMC12892260

[82] Anand RP Layer JV Heja D Hirose T Lassiter G Firl DJ. Design and testing of a humanized porcine donor for xenotransplantation. Nature. (2023) 622(7982):393–401. 10.1038/s41586-023-06594-4 37821590 PMC10567564

[83] Bongoni AK Kiermeir D Schnider J Jenni H Garimella P Bähr A. Transgenic expression of human CD46 on porcine endothelium: effect on coagulation and fibrinolytic cascades during *ex vivo* human-to-pig limb xenoperfusions. Transplantation. (2015) 99(10):2061–9. 10.1097/TP.0000000000000746 25965410

[84] McGregor CGA Ricci D Miyagi N Stalboerger PG Du Z Oehler EA. Human CD55 expression blocks hyperacute rejection and restricts complement activation in gal knockout cardiac xenografts. Transplantation. (2012) 93(7):686–92. 10.1097/TP.0b013e3182472850 22391577 PMC3314133

[85] Lei Y Wolf-van Buerck L Honarpisheh M Zhang Y Schwinzer R Petersen B. Neonatal islets from human PD-L1 transgenic pigs reduce immune cell activation and cellular rejection in humanized nonobese diabetic-scid IL2r*γ*null mice. Am J Transplant. (2024) 24(1):20–9. 10.1016/j.ajt.2023.08.026 37659605

[86] Schmalkuche K Rother T Besli S Schwinzer R Blasczyk R Peterson B. Human PD-L1 overexpression decreases xenogeneic human T-cell immune responses towards porcine kidneys. Front Immunol. (2024) 15:1279050. 10.3389/fimmu.2024.1279050 38352884 PMC10861674

[87] Singh AK Chan JL DiChiacchio L Hardy NL Corcoran PC Lewis BGT. Cardiac xenografts show reduced survival in the absence of transgenic human thrombomodulin expression in donor pigs. Xenotransplantation. (2019) 26(2):e12465. 10.1111/xen.12465 30290025 PMC6450784

[88] Kim J Kim M Hwang DG Shim IK Kim SC Jang J. Pancreatic tissue-derived extracellular matrix bioink for printing 3D cell-laden pancreatic tissue constructs. J Vis Exp. (2019) (154):. 10.3791/60434 31885383

[89] Wang D Guo Y Zhu J Liu F Xue Y Huang Y. Hyaluronic acid methacrylate/pancreatic extracellular matrix as a potential 3D printing bioink for constructing islet organoids. Acta Biomater. (2023) 165:86–101. 10.1016/j.actbio.2022.06.036 35803504

[90] Jeong W Perrier Q Rengaraj A Byers L Gonzalez GC Peveri E. Scalable 3D Bioprinting of Human Islets in a Pancreatic Decellularized Extracellular Matrix-Enriched Bioink for Beta-Cell Replacement Therapy. bioRxiv. 2025:2025.06.06.658360. 10.1101/2025.06.06.658360 42570781

[91] Idaszek J Volpi M Paradiso A Nguyen Quoc M Górecka Ż Klak M. Alginate-based tissue-specific bioinks for multi-material 3D-bioprinting of pancreatic islets and blood vessels: a step towards vascularized pancreas grafts. Bioprinting. (2021) 24:e00163. 10.1016/j.bprint.2021.e00163

[92] Kim M Cho S Hwang DG Shim IK Kim SC Jang J. Bioprinting of bespoke islet-specific niches to promote maturation of stem cell-derived islets. Nat Commun. (2025) 16(1):1430. 10.1038/s41467-025-56665-5 39920133 PMC11805982

[93] Edri S Newman Frisch A Safina D Machour M Zavin J Landsman L. 3D Bioprinting of multicellular stem cell-derived constructs to model pancreatic cell differentiation. Adv Funct Mater. (2024) 34(30):2315488. 10.1002/adfm.202315488

[94] Shizukuda Y Tang S Yokota R Ware JA. Vascular endothelial growth factor-induced endothelial cell migration and proliferation depend on a nitric oxide-mediated decrease in protein kinase C activity. Circ Res. (1999) 85(3):247–56. 10.1161/01.res.85.3.247 10436167

[95] Wang S Li X Parra M Verdin E Bassel-Duby R Olson EN. Control of endothelial cell proliferation and migration by VEGF signaling to histone deacetylase 7. Proc. Natl. Acad. Sci. (2008) 105(22):7738–43. 10.1073/pnas.0802857105 18509061 PMC2409381

[96] Mao D Zhu M Zhang X Rong M Yang X Ke T. A macroporous heparin-releasing silk fibroin scaffold improves islet transplantation outcome by promoting islet revascularisation and survival. Acta Biomater. (2017) 59:210–20. 10.1016/j.actbio.2017.06.039 28666883

[97] Brissova M Shostak A Shiota M Masakazu W Peter O Poffenberger G. Pancreatic islet production of vascular endothelial growth factor-A is essential for islet vascularization, revascularization, and function. Diabetes. (2006) 55(11):2974–85. 10.2337/DB06-0690 17065333

[98] Phelps EA Templeman KL Thulé PM García AJ. Engineered VEGF-releasing PEG–MAL hydrogel for pancreatic islet vascularization. Drug Deliv Transl Res. (2013) 5(2):125–36. 10.1007/S13346-013-0142-2 PMC4366610 25787738

[99] Kang S Park HS Jo A Hong SH Lee HN Lee YY. Endothelial progenitor cell cotransplantation enhances islet engraftment by rapid revascularization. Diabetes. (2012) 61(4):866–76. 10.2337/DB10-1492 22362173 PMC3314353

[100] Fransson M Brännström J Duprez I Essand M Le Blanc K Korsgren O. Mesenchymal stromal cells support endothelial cell interactions in an intramuscular islet transplantation model. Regen Med Res. (2015) 30:1. 10.1186/s40340-015-0010-9 PMC4589952 26430512

[101] Zacharovová K Berková Z Girman P Saudek F. Adipose tissue-derived mesenchymal stem cells promote the vascularization of pancreatic islets transplanted into decellularized pancreatic skeletons. Transpl Immunol. (2024) 86:102106. 10.1016/j.trim.2024.102106 39128811

[102] Pepper AR Gala-Lopez B Pawlick R Merani S Kin T Shapiro AMJ. A prevascularized subcutaneous device-less site for islet and cellular transplantation. Nat Biotechnol. (2015) 33(5):518–23. 10.1038/nbt.3211 25893782

[103] Liu Y Yang M Cui Y Yao Y Liao M Yuan H. A novel prevascularized tissue-engineered chamber as a site for allogeneic and xenogeneic islet transplantation to establish a bioartificial pancreas. PLoS One. (2020) 15(12):e0234670. 10.1371/JOURNAL.PONE.0234670 33270650 PMC7714105

[104] Hussey AJ Winardi M Han XL Thomas GPL Penington AJ Morrison WA. Seeding of pancreatic islets into prevascularized tissue engineering chambers. Tissue Eng Part A. (2009) 15(12):3823–33. 10.1089/TEN.TEA.2008.0682 19558221

[105] Forster NA Penington AJ Hardikar AA Palmer JA Hussey A Tai J. A prevascularized tissue engineering chamber supports growth and function of islets and progenitor cells in diabetic mice. Islets. (2011) 3(5):271–83. 10.4161/ISL.3.5.15942 21847009

[106] Vlahos AE Talior-Volodarsky I Kinney SM Sefton MV. A scalable device-less biomaterial approach for subcutaneous islet transplantation. Biomaterials. (2021) 269:120499. 10.1016/j.biomaterials.2020.120499 33168223

[107] Paez-Mayorga J Campa-Carranza JN Capuani S Hernandez N Liu HC Chua CYX. Implantable niche with local immunosuppression for islet allotransplantation achieves type 1 diabetes reversal in rats. Nat Commun. (2022) 13(1):7951. 10.1038/s41467-022-35629-z 36572684 PMC9792517

[108] Wei Z Lei M Wang Y Xie Y Xie X Lan D. Hydrogels with tunable mechanical plasticity regulate endothelial cell outgrowth in vasculogenesis and angiogenesis. Nat Commun. (2023) 14(1):8307. 10.1038/s41467-023-43768-0 38097553 PMC10721650

[109] Hao D Liu R Gao K He C He S Zhao C. Developing an injectable nanofibrous extracellular matrix hydrogel with an integrin *α*v*β*3 ligand to improve endothelial cell survival, engraftment and vascularization. Front Bioeng Biotechnol. (2020) 8:544722. 10.3389/FBIOE.2020.00890 PMC7403189 32850742

[110] Zhang M Zhao F Zhu Y Brouwer LA Van Der Veen H Burgess JK. Physical properties and biochemical composition of extracellular matrix-derived hydrogels dictate vascularization potential in an organ-dependent fashion. ACS Appl Mater Interfaces. (2024) 16(23):29930–45. 10.1021/acsami.4c05864 38819955 PMC11181272

[111] Dufrane D Goebbels RM Gianello P. Alginate macroencapsulation of pig islets allows correction of streptozotocin-induced diabetes in primates up to 6 months without immunosuppression. Transplantation. (2010) 90(10):1054–62. 10.1097/tp.0b013e3181f6e267 20975626

[112] Bochenek MA Veiseh O Vegas AJ McGarrigle JJ Qi MME Omami M. Alginate encapsulation as long-term immune protection of allogeneic pancreatic islet cells transplanted into the omental Bursa of macaques. Nat Biomed Eng. (2018) 2(11):810–21. 10.1038/s41551-018-0275-1 30873298 PMC6413527

[113] Alagpulinsa DA Cao JJL Driscoll RK Sîrbulescu R Penson M Sremac M. Alginate-microencapsulation of human stem cell–derived *β* cells with CXCL12 prolongs their survival and function in immunocompetent mice without systemic immunosuppression. Am J Transplant. (2019) 19(7):1930–40. 10.1111/ajt.15308 30748094

[114] Pellicciaro M Vella I Lanzoni G Tisone G Ricordi C. The greater omentum as a site for pancreatic islet transplantation. CellR4 Repair Replace Regen Reprogram. (2017) 5(3):e2410. 33834082 PMC8025931

[115] Laschewsky A Rosenhahn A. Molecular design of zwitterionic polymer interfaces: searching for the difference. Langmuir. (2018) 35(5):1056–71. 10.1021/acs.langmuir.8b01789 30048142

[116] Liu Q Chiu A Wang LH An D Zhong M Smink AM. Zwitterionically modified alginates mitigate cellular overgrowth for cell encapsulation. Nat Commun. (2019) 10(1):5262. 10.1038/s41467-019-13238-7 31748525 PMC6868136

[117] Wang LH Ernst AU An D Datta A Kumar E Boris K. A bioinspired scaffold for rapid oxygenation of cell encapsulation systems. Nat Commun. (2021) 12(1):5846. 10.1038/s41467-021-26126-w 34615868 PMC8494927

[118] Tomei AA Manzoli V Fraker CA Giraldo J Velluto D Najjar M. Device design and materials optimization of conformal coating for islets of langerhans. Proc Natl Acad Sci U S A. (2014) 111(29):10514–9. 10.1073/PNAS.1402216111 24982192 PMC4115512

[119] Stock AA Manzoli V De Toni T Abreu MM Poh Y Ye L. Conformal coating of stem cell-derived islets for *β* cell replacement in type 1 diabetes. Stem Cell Rep. (2020) 14(1):91–104. 10.1016/j.stemcr.2019.11.004 PMC6962554 31839542

[120] Frei AW Li Y Jiang K Buchwald P Stabler CL. Local delivery of fingolimod from three-dimensional scaffolds impacts islet graft efficacy and microenvironment in a murine diabetic model. J Tissue Eng Regen Med. (2018) 12(2):393–404. 10.1002/term.2464 28486786

[121] Jiang K Weaver JD Li Y Chen X Liang J Stabler CL. Local release of dexamethasone from macroporous scaffolds accelerates islet transplant engraftment by promotion of anti-inflammatory M2 macrophages. Biomaterials. (2017) 114:71–81. 10.1016/j.biomaterials.2016.11.004 27846404

[122] Wang X Wang K Yu M Velluto D Hong X Wang B. Engineered immunomodulatory accessory cells improve experimental allogeneic islet transplantation without immunosuppression. Sci Adv. (2022) 8(29):71. 10.1126/SCIADV.ABN0071 PMC9307254 35867788

[123] Barra JM Kozlovskaya V Burnette KLS Banerjee RR Fraker CA Kharlampieva E. Localized cytotoxic T cell–associated antigen 4 and antioxidant islet encapsulation alters macrophage signaling and induces regulatory and anergic T cells to enhance allograft survival. Am J Transplant. (2023) 23(4):498–511. 10.1016/j.ajt.2023.01.007 36731781 PMC10291560

[124] Hu X Gattis C Olroyd AG Friera AM White K Chi Y. Human hypoimmune primary pancreatic islets avoid rejection and autoimmunity and alleviate diabetes in allogeneic humanized mice. Sci Transl Med. (2023) 15(691):eadg5794. 10.1126/scitranslmed.adg5794 37043559

[125] Longnecker DS. Anatomy and histology of the pancreas. Pancreapedia: the Exocrine Pancreas Knowledge Base. Published Online. (2021). 10.3998/PANC.2021.01

[126] Wang YJ Golson ML Schug J Traum D Liu C Vivek K. Single-Cell mass cytometry analysis of the human endocrine pancreas. Cell Metab. (2016) 24(4):616–26. 10.1016/j.cmet.2016.09.007 27732837 PMC5123805

[127] Rezania A Bruin JE Arora P Rubin A Batushansky I Asadi A. Reversal of diabetes with insulin-producing cells derived *in vitro* from human pluripotent stem cells. Nat Biotechnol. (2014) 32(11):1121–33. 10.1038/nbt.3033 25211370

[128] Maxwell KG Millman JR. Applications of iPSC-derived beta cells from patients with diabetes. Cell Rep Med. (2021) 2(4):100238. 10.1016/J.XCRM.2021.100238 33948571 PMC8080107

[129] Nostro MC Keller G. Generation of beta cells from human pluripotent stem cells: potential for regenerative medicine. Semin Cell Dev Biol. (2012) 23(6):701–10. 10.1016/J.SEMCDB.2012.06.010 22750147 PMC4400853

[130] Miller J Perrier Q Rengaraj A Bowlby J Byers L Peveri E. State of the art of bioengineering approaches in Beta-cell replacement. Curr Transplant Rep. (2025) 12(1):17. 10.1007/S40472-025-00470-y 40342868 PMC12055624

[131] Wieland FC Sthijns MMJPE Geuens T Van Blitterswijk CA Lapointe VLS. The role of alpha cells in the self-assembly of bioengineered islets. Tissue Eng Part A. (2021) 27(15-16):1055–63. 10.1089/ten.TEA.2020.0080 33076775 PMC8392094

[132] Lee I. Human pancreatic islets develop through fusion of distinct *β* and *α*/*δ* islets. Dev Growth Differ. (2016) 58(8):635–40. 10.1111/DGD.12308 27530443

[133] Citro A Ott HC. Can we Re-engineer the endocrine pancreas? Curr Diab Rep. (2018) 18(11):122. 10.1007/S11892-018-1072-7 30280279

[134] Witjas FMR van den Berg BM van den Berg CW Engelse MA Rabelink TJ. Concise review: the endothelial cell extracellular matrix regulates tissue homeostasis and repair. Stem Cells Transl Med. (2019) 8(4):375–82. 10.1002/SCTM.18-0155 30537441 PMC6431685

[135] Geng A Yuan S Yu QC Zeng YA. The role of endothelial cells in pancreatic islet development, transplantation and culture. Front Cell Dev Biol. (2025) 13:1558137. 10.3389/FCELL.2025.1558137 40330424 PMC12052768

[136] Peterson QP Veres A Chen L Slama MQ Kenty JHR Hassoun S. A method for the generation of human stem cell-derived alpha cells. Nat Commun. (2020) 11(1):2241. 10.1038/s41467-020-16049-3 32382023 PMC7205884

[137] Yabe SG Fukuda S Nishida J Takeda F Nashiro K Okochi H. Efficient induction of pancreatic alpha cells from human induced pluripotent stem cells by controlling the timing for BMP antagonism and activation of retinoic acid signaling. PLoS One. (2021) 16(1):e0245204. 10.1371/journal.pone.0245204 33428669 PMC7799802

[138] Kaestner KH Powers AC Naji A Atkinson MA. NIH Initiative to improve understanding of the pancreas, islet, and autoimmunity in type 1 diabetes: the human pancreas analysis program (HPAP). Diabetes. (2019) 68(7):1394–402. 10.2337/DB19-0058 31127054 PMC6609987

[139] Di Mauro M Papalia GG Le Moli RR Nativo BB Nicoletti FF Lunetta MM. Effect of octreotide on insulin requirement, hepatic glucose production, growth hormone, glucagon and c-peptide levels in type 2 diabetic patients with chronic renal failure or normal renal function. Diabetes Res Clin Pract. (2001) 51(1):45–50. 10.1016/s0168-8227(00)00203-5 11137181

[140] Gerich JE Schultz TA Lewis SB Karam JH. Clinical evaluation of somatostatin as a potential adjunct to insulin in the management of diabetes mellitus. Diabetologia. (1977) 13(5):537–44. 10.1007/bf01234510 908478

[141] Zhang T Wang N Liao Z Chen J Meng H Lin H. A differentiation protocol for generating pancreatic delta cells from human pluripotent stem cells. Front Cell Dev Biol. (2024) 12:1490040. 10.3389/fcell.2024.1490040 39493348 PMC11527672

[142] Chen L Wang N Zhang T Zhang W Meng H Chen J. Directed differentiation of pancreatic *δ* cells from human pluripotent stem cells. Nat Commun. (2024) 15(1):6344. 10.1038/s41467-024-50611-7 39068220 PMC11283558

[143] Sundler F Håkanson R Larsson LI. Ontogeny of rat pancreatic polypeptide (PP) cells. Cell Tissue Res. (1977) 178(3):303–6. 10.1007/BF00218694 321125

[144] Stefan Y Orci L Malaisse-Lagae F Perrelet A Patel Y Unger RH. Quantitation of endocrine cell content in the pancreas of nondiabetic and diabetic humans. Diabetes. (1982) 31(8):694–700. 10.2337/DIAB.31.8.694 6131002

[145] Clark A Wells CA Buley ID Cruickshank JK Vanhegan RI Matthews DR. Islet amyloid, increased A-cells, reduced B-cells and exocrine fibrosis: quantitative changes in the pancreas in type 2 diabetes. Diabetes Res. (1988) 9(4):151–9. 3073901

[146] Kou X Liu J Wang D Yu M Li C Lu L. Exocrine pancreas regeneration modifies original pancreas to alleviate diabetes in mouse models. Sci Transl Med. (2022) 14(656):eabg9170. 10.1126/scitranslmed.abg9170 35921475

[147] Pan FC Bankaitis ED Boyer D Xu X Van de Casteele M Magnuson MA. Spatiotemporal patterns of multipotentiality in Ptf1a-expressing cells during pancreas organogenesis and injury-induced facultative restoration. Development. (2013) 140(4):751–64. 10.1242/dev.090159 23325761 PMC3557774

[148] Baeyens L De Breuck S Lardon J Mfopou JK Rooman I Bouwens L. *In vitro* generation of insulin-producing beta cells from adult exocrine pancreatic cells. Diabetologia. (2004) 48(1):49–57. 10.1007/S00125-004-1606-1 15616797

[149] Yew KH Hembree M Prasadan K Preuett B McFall C Benjes C. Cross-talk between bone morphogenetic protein and transforming growth factor-*β* signaling is essential for exendin-4-induced insulin-positive differentiation of AR42J cells. J Biol Chem. (2005) 280(37):32209–17. 10.1074/jbc.m505465200 16020542

[150] Zhou J Wang X Pineyro MA Egan JM. Glucagon-like peptide 1 and exendin-4 convert pancreatic AR42J cells into glucagon- and insulin-producing cells. Diabetes. (1999) 48(12):2358–66. 10.2337/DIABETES.48.12.2358 10580424

[151] Smink AM Rodriquez S Li S Ceballos B Corrales N Alexander M. Successful islet transplantation into a subcutaneous polycaprolactone scaffold in mice and pigs. Transplant Direct. (2022) 9(1):E1417. 10.1097/TXD.0000000000001417 36591328 PMC9788983

[152] French A Hollister-Lock J Sullivan BA Stas E Hwa AJ Weir GC. Enhancement of subcutaneous islet transplant performance by collagen 1 gel. Cell Transplant. (2024) 33:9636897241283728. 10.1177/09636897241283728 39361612 PMC11457190

[153] Goh SK Bertera S Olsen P Candiello JE Halfter W Uechi G. Perfusion-decellularized pancreas as a natural 3D scaffold for pancreatic tissue and whole organ engineering. Biomaterials. (2013) 34(28):6760–72. 10.1016/J.BIOMATERIALS.2013.05.066 23787110 PMC3748589

[154] Citro A Neroni A Pignatelli C Campo F Policardi M Monieri M. Directed self-assembly of a xenogeneic vascularized endocrine pancreas for type 1 diabetes. Nat Commun. (2023) 14(1):878. 10.1038/s41467-023-36582-1 36797282 PMC9935529

[155] Takahashi Y Sekine K Kin T Takebe T Taniguchi H. Self-Condensation culture enables vascularization of tissue fragments for efficient therapeutic transplantation. Cell Rep. (2018) 23(6):1620–9. 10.1016/j.celrep.2018.03.123 29742420 PMC8289710

[156] Deng H Zhang A Pang DRR Xi Y Yang Z Matheson R. Bioengineered omental transplant site promotes pancreatic islet allografts survival in non-human primates. Cell Rep Med. (2023) 4(3):100959. 10.1016/J.XCRM.2023.100959 36863336 PMC10040375

[157] Oppler SH Stone LLH Leishman DJ Janecek JL Moore MEG Rangarajan P. A bioengineered artificial interstitium supports long-term islet xenograft survival in nonhuman primates without immunosuppression. Sci Adv. (2024) 10(1):4919. 10.1126/SCIADV.ADI4919 PMC10776017 38181083

[158] Barkai U Rotem A Vos P. Survival of encapsulated islets: more than a membrane story. World J Transplant. (2016) 6(1):69–90. 10.5500/WJT.V6.I1.69 27011906 PMC4801806

[159] Carlsson PO Espes D Sedigh A Rotem A Zimerman B Grinberg H. Transplantation of macroencapsulated human islets within the bioartificial pancreas *β*Air to patients with type 1 diabetes mellitus. Am J Transplant. (2018) 18(7):1735–44. 10.1111/ajt.14642 29288549 PMC6055594

[160] Kumagai-Braesch M Jacobson S Mori H Jia X Takahasi T Wernerson A. The theracyte^TM^ device protects against islet allograft rejection in immunized hosts. Cell Transplant. (2013) 22(7):1137–46. 10.3727/096368912X657486 23043940

[161] Sernova Corp. Sernova Provides Clinical Update on U.S. Phase I/II Cell Pouch Trial for Type 1 Diabetes. London, Ontario, Canada: Sernova Corp. (2020). Available online at: https://sernova.com/press_releases/sernova-provides-clinical-update-on-u-s-phase-i-ii-cell-pouch-trial-for-type-1-diabetes/ (Accessed December 7, 2025).

[162] Sernova Corp. Sernova Biotherapeutics Provides Update on Phase 1/2 Clinical Trial of Cell PouchTM Bio-Hybrid Organ for Treatment of Type 1 Diabetes. London, Ontario, Canada: Sernova Corp (2025). Available online at: https://sernova.com/press_releases/sernova-biotherapeutics-provides-update-on-phase-1-2-clinical-trial-of-cell-pouch-bio-hybrid-organ-for-treatment-of-type-1-diabetes/ (Accessed December 07, 2025).

[163] Baidal DA Ricordi C Berman DM Alvarez A Padilla N Ciancio G. Bioengineering of an intraabdominal endocrine pancreas. N Engl J Med. (2017) 376(19):1887–9. 10.1056/NEJMc1613959 28489987 PMC5572072

[164] Shapiro AMJ Thompson D Donner TW Bellin MD Hsueh W Pettus J. Insulin expression and C-peptide in type 1 diabetes subjects implanted with stem cell-derived pancreatic endoderm cells in an encapsulation device. Cell Rep Med. (2021) 2(12):100466. 10.1016/J.XCRM.2021.100466 35028608 PMC8714853

[165] Keymeulen B De Groot K Jacobs-Tulleneers-Thevissen D Thompson DM Bellin MD Kroon EJ. Encapsulated stem cell–derived *β* cells exert glucose control in patients with type 1 diabetes. Nat Biotechnol. (2024) 42(10):1507–14. 10.1038/s41587-023-02055-5 38012450 PMC11471599

[166] Reichman TW Markmann JF Odorico J Witkowski P Fung JJ Wijkstrom M. Stem cell–derived, fully differentiated islets for type 1 diabetes. N Engl J Med. (2025) 393(9):858–68. 10.1056/NEJMOA2506549 40544428

[167] Hiyoshi H Sakuma K Asano S Napier SC Konagaya S Mochida T. Identification and removal of unexpected proliferative off-target cells emerging after iPSC-derived pancreatic islet cell implantation. Proc Natl Acad Sci U S A. (2024) 121(16):e2320883121. 10.1073/PNAS.2320883121 38598342 PMC11032438

[168] Zhong C Liu M Pan X Zhu H. Tumorigenicity risk of iPSCs *in vivo*: nip it in the bud. Precis Clin Med. (2022) 5(1):pbac004. 10.1093/PCMEDI/PBAC004 35692443 PMC9026204

[169] Wang Y Chen Y McGarrigle J Cook J Rios PD La Monica G. Cell therapy for T1D beyond BLA: gearing up toward clinical practice. Diabetes Ther. (2025) 16(6):1125–38. 10.1007/S13300-025-01732-9 40214896 PMC12085407

[170] Nakayama M Moriya Y Ueno H Watanabe T Hirabayashi H Yamamoto S. Considerations in biodistribution evaluation of iPSC-derived cell therapy: a pancreatic islet cell case study. Mol Ther Methods Clin Dev. (2025) 33(3):101538. 10.1016/j.omtm.2025.101538 40777725 PMC12329530

[171] U.S. Food & Drug Administration. Guidance for Industry: Considerations for Allogeneic Pancreatic Islet Cell Products. Silver Spring, MD: U.S. Food & Drug Administration (2009). Accessed March 11, 2025. Available online at: https://www.fda.gov/regulatory-information/search-fda-guidance-documents/considerations-allogeneic-pancreatic-islet-cell-products?

[172] U.S Food & Drug Administration. Guidance for Industry: Guidance for Human Somatic Cell Therapy and Gene Therapy. Silver Spring, MD: U.S. Food & Drug Administration (1998). Accessed December 7, 2025. Available online at: https://www.fda.gov/files/vaccines%2C%20blood%20%26%20biologics/published/Guidance-for-Industry–Guidance-for-Human-Somatic-Cell-Therapy-and-Gene-Therapy.pdf?

[173] Benton K. US FDA regulatory framework for cellular therapy products. In: Global Regulatory Perspectives Workshop. Silver Spring, MD: U.S. Food & Drug Administration (2013). Accessed December 7, 2025. Available online at: https://cdn.ymaws.com/my.isctglobal.org/resource/resmgr/resources_2013presentations/benton.k.grp2013.pdf?

[174] Witkowski P. ASTS Statement on Islet Cell Transplantation Regulations. Arlington, VA: American Society of Transplant Surgeons (2020). Accessed December 7, 2025. Available online at: https://www.asts.org/docs/default-source/position-statements/asts-statement-on-islet-cell-transplantation-regulations.pdf?sfvrsn=7a614ed3_3&

[175] Witkowski P Odorico J Pyda J Anteby R Sratta RJ Schrope BA. Arguments against the requirement of a biological license application for human pancreatic islets: the position statement of the islets for US collaborative presented during the fda advisory committee meeting. J Clin Med. (2021) 10(13):2878. 10.3390/JCM10132878/S1 34209541 PMC8269003

[176] Shapiro AMJ. Islet transplantation – the Canadian perspective. CellR4 Repair Replace Regen Reprogram. (2019) 7:e2799. 10.32113/cellr4_201911_2799 31828180 PMC6905502

[177] Berney T Andres A Bellin MD de Koning EJP Johnson PRV Kay TWH. A worldwide survey of activities and practices in clinical islet of langerhans transplantation. Transpl Int. (2022) 35:10507. 10.3389/TI.2022.10507 36033644 PMC9402897

[178] Xiang Y Bernards N Hoang B Zheng J Matsuura N. Perfluorocarbon nanodroplets can reoxygenate hypoxic tumors *in vivo* without carbogen breathing. Nanotheranostics. (2019) 3(2):135–44. 10.7150/NTNO.29908 31008022 PMC6470341

[179] Zhou Z Zhang B Wang H Yuan A Hu Y Wu J. Two-stage oxygen delivery for enhanced radiotherapy by perfluorocarbon nanoparticles. Theranostics. (2018) 8(18):4898–911. 10.7150/THNO.27598 30429876 PMC6217071

[180] Nanduru A Egwom P Kiss A Cresswell G Johnson J Unger EC. 5 Immunomodulation Of tumor microenvironment in triple negative breast cancer by reversing hypoxia with NanO2, a novel oxygen delivery agent. J Immunother Cancer. (2025) 13(Suppl 2):A5. 10.1136/JITC-2025-SITC2025.0005

